# Signaling Overview of Plant Somatic Embryogenesis

**DOI:** 10.3389/fpls.2019.00077

**Published:** 2019-02-07

**Authors:** Hugo A. Méndez-Hernández, Maharshi Ledezma-Rodríguez, Randy N. Avilez-Montalvo, Yary L. Juárez-Gómez, Analesa Skeete, Johny Avilez-Montalvo, Clelia De-la-Peña, Víctor M. Loyola-Vargas

**Affiliations:** ^1^Unidad de Bioquímica y Biología Molecular de Plantas, Centro de Investigación Científica de Yucatán, Mérida, Mexico; ^2^Unidad de Biotecnología, Centro de Investigación Científica de Yucatán, Mérida, Mexico

**Keywords:** differentiation, growth regulators, signaling, somatic embryogenesis, totipotency, transcription factors

## Abstract

Somatic embryogenesis (SE) is a means by which plants can regenerate bipolar structures from a somatic cell. During the process of cell differentiation, the explant responds to endogenous stimuli, which trigger the induction of a signaling response and, consequently, modify the gene program of the cell. SE is probably the most studied plant regeneration model, but to date it is the least understood due to the unclear mechanisms that occur at a cellular level. In this review, the authors seek to emphasize the importance of signaling on plant SE, highlighting the interactions between the different plant growth regulators (PGR), mainly auxins, cytokinins (CKs), ethylene and abscisic acid (ABA), during the induction of SE. The role of signaling is examined from the start of cell differentiation through the early steps on the embryogenic pathway, as well as its relation to a plant’s tolerance of different types of stress. Furthermore, the role of genes encoded to transcription factors (TFs) during the embryogenic process such as the *LEAFY COTYLEDON (LEC)*, *WUSCHEL (WUS)*, *BABY BOOM (BBM)* and *CLAVATA (CLV*) genes, Arabinogalactan-proteins (AGPs), *APETALA 2 (AP2)* and epigenetic factors is discussed.

## Introduction

Higher plant embryogenesis is divided conceptually into two distinct phases: early morphogenetic processes that give rise to embryonic cell types, tissues, and organ systems, and late maturation events that allow the fully developed embryo to enter a desiccated and metabolically quiescent state (West and Harada, 1993; Goldberg et al., 1994). Embryogenesis is the process by which embryo formation is initiated, either from a zygote (zygotic embryogenesis, ZE) or from somatic cells (somatic embryogenesis, SE). ZE is carried out after the fusion of gametes. However, the formation of asexual embryos can be induced *in vitro* from cells that come from an explant of vegetal tissue (Loyola-Vargas and Ochoa-Alejo, 2016). The SE process also occurs in nature. Under certain environmental conditions such as heat and drought, the plant Kalanchoë produces, around their leaves, small bipolar structures, which develop later in plantlets (Garcês and Sinha, 2009). There are several other paths leading to the formation of an embryo. For instance, apomictic embryogenesis takes place in the seed primordium (ovule) and the embryos produced are genetically identical to the mother plant. Microspores can also produce embryos, and the cells of the suspensor can change their identity to embryogenic cells when the original embryo loses its capacity to develop (Radoeva and Weijers, 2014).

Somatic embryogenesis represents a complete model of totipotency and involves the action of a complex signaling network, as well as the reprogramming of gene expression patterns that are regulated in a specific way. This gene regulation usually is in response to exogenous stimuli produced by the use of plant growth regulators (PGR) or certain stress conditions, mainly low or high temperature, heavy metals, osmotic shock or drought (Nic-Can et al., 2016). The induction of SE *in vitro* can be accomplished through two pathways. When SE is direct, somatic embryos are formed at the edge of an explant; when it is indirect, SE occurs through the proliferation of a disorganized and dedifferentiated tissue called callus (Quiroz-Figueroa et al., 2006).

Somatic embryogenesis has several biological and scientific advantages. For instance, it has the potential for the improvement of plants of commercial importance, as well as for the study of the genetic and physiological changes that are related to the fate of a plant cell. Until now, most studies have examined the mechanisms involved in the induction of the SE process using model plant species, such as carrot, alfalfa, corn, and rice. However, other species, such as *Arabidopsis thaliana* and *Gossypium hirsutum*, have been used to study the signaling pathways of the PGR action leading to the development of plant cells (Zhou et al., 2016).

## Early Somatic Embryogenesis

Once the somatic cells are induced to generate cells with embryogenic capacity, the new cells can form structures capable of regenerating a complete plant. System suspensors are very noticeable in gymnosperm somatic embryos. However, in many angiosperms, suspensors are either absent or strongly reduced due to the absence of the hypophyseal cell (Smertenko and Bozhkov, 2014).

**FIGURE 1:**
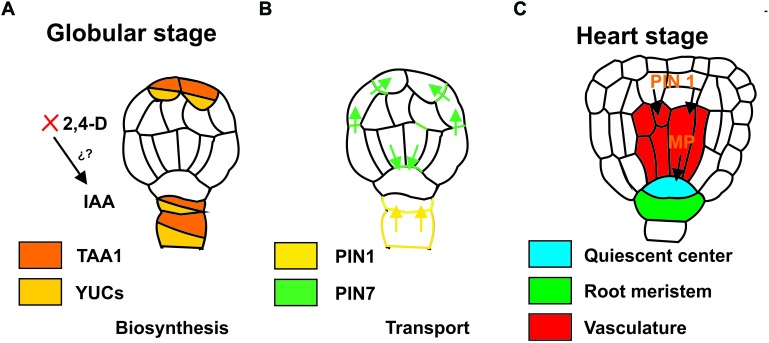
Auxin biosynthesis during the induction of somatic embryogenesis. **(A)** Auxin transport during the development of somatic embryos, globular stage **(B)** and heart stage **(C)**. Colors indicate the localization of the expression of the genes. IAA, indole-3-acetic acid; 2, 4-D, 2, 4-Dichloroacetic acid; *TAA1*, *TRYPTOPHAN AMINOTRANSFERASE OF ARABIDOPSIS 1*; *YUC*, *YUCCA*; *PIN*, *PIN-FORMED*; MP, monopteros.

It is unclear how cells initiate embryo formation. Nonetheless, it has been established that an irregular distribution of auxins must be established to initiate embryo formation. This asymmetrical auxin distribution results from differential transport (Márquez-López et al., 2018; Figure 1). In the case of ZE, an asymmetric cell division occurs, whereas in SE this is often not observed (Toonen et al., 1994). An asymmetric mitotic division of the zygote produces two different cells: one cell gives rise to the suspensor and the other to the embryo proper. At the octant and globular stage, protoderm formation and primordial initiation takes place (Dodeman et al., 1997). The differential transport and asymmetrical auxin distribution continue during these stages, giving rise to the different tissues that will form the embryo. The transportation and accumulation of auxin produce the interaction with other factors, such as cytokinins (CKs), which leads to the expression of specific genes (Quiroz-Figueroa et al., 2002).

## Stages of Embryo Development

Although there is a morphological resemblance between somatic and zygotic embryos, their development is distinctive based on plant classification (angiosperms and gymnosperms). It is considered that zygotic embryos are nourished via the phloem tissue, whereas somatic embryos use an exogenous supply of carbohydrates and their morphological stages occur without vascular tissue connection (Pila Quinga et al., 2018).

Theoretically, plant development can be divided into two different phases: (1) embryogenesis *sensu stricto*, which begins with the formation of the zygote and concludes at the cotyledonary stage, and (2) the maturation of the seed (Dodeman et al., 1997). The somatic and zygotic embryo developmental stages are divided into two main metabolic phases. The first is at a morphogenetic level, where the meristem activity is triggered at a physiological level and the process of growth, storage and maturation is initiated. The second is a metabolic stage that is characterized by biochemical activities and the preparation for desiccation to complete the seed formation process (Harada and Kwong, 2002; Pila Quinga et al., 2018). In this last phase, somatic embryos achieve both morphological and physiological maturity, which guarantees satisfactory post-embryonic performance. Therefore, the conversion potential is considered to be programmed during embryo maturation. However, somatic embryos do not require desiccation (Smertenko and Bozhkov, 2014).

Somatic embryo development involves similar stages to ZE, such as the globular-shaped, heart-shaped, torpedo-shaped, and cotyledonal stages in the case of dicotyledonous species (Winkelmann, 2016), and globular, scutellar, and coleoptile stages in the case of monocotyledonous species (Zhao et al., 2017). Once the somatic embryos reach the cotyledonary stage, they initiate a shoot meristem, and seedling growth begins (Yang and Zhang, 2010).

## Factors That Induce Somatic Embryogenesis

Understanding the physiological and molecular mechanisms by which the induction (direct or indirect) of SE occurs is a crucial step for its manipulation (Grzyb et al., 2018). Several factors can induce SE. The conditions of the culture medium, the high concentrations of PGRs, and the wounding of explant are other types of stress that can cause plant cells to change their cellular and molecular programs. The type of explant, the age and the genotype of the mother plant, the physiological conditions of the incubation, and the cellular density in the case of suspension cultures, as well as the generation of homogeneous cell aggregates, are factors that must be considered in order to produce the acquisition of embryogenic potential (Pencik et al., 2015; Loyola-Vargas and Ochoa-Alejo, 2016).

The source of nitrogen, as well as its concentration in the culture medium, has been shown to be an essential element for the induction of SE (Reinert et al., 1967). In different plant species, such as *Cucurbita pepo* (Pencik et al., 2015), *Medicago sativa* (Walker and Sato, 1981), *Coffea arabica* (Fuentes-Cerda et al., 2001), and *Daucus carota* (Kamada and Harada, 1979), it has been determined that both nitrate and ammonium content in the culture medium have a significant effect on the response of the explants to the induction of SE. It has been proposed that stress is the switch that stimulates cellular reprogramming toward an embryogenic path (Nic-Can et al., 2016). However, the mechanism by which the nitrogen sources participate in the induction of embryogenic potential remains unknown.

## The Role of Plant Growth Regulators During the Induction of Somatic Embryogenesis

In plant culture systems, the addition of PGR to the culture medium plays an important role in inducing cell differentiation, in particular during the induction of SE. Most of the SE process depends on the concentration and kind of PGR used for each culture. Different plant species, such as *C. canephora* (Márquez-López et al., 2018), *A. thaliana* (Grzybkowska et al., 2018), and *Musa spp*. (Awasthi et al., 2017) responded successfully to the SE induction using different explants, conditions, and concentrations of PGR.

Many species that are able to produce somatic embryos from cell suspension cultures require the addition of auxins in the culture medium. The use of 2, 4-dichloroacetic acid (2, 4-D) has an essential role in the induction of SE and the initial stages of development of the somatic embryos (Nic-Can and Loyola-Vargas, 2016). For example, the productivity for embryogenic date palm crops increased 20 times by adding a low concentration of 2, 4-D (Abohatem et al., 2017). The use of auxins modified their endogenous metabolism in a significant way; for example, in carrots, the use of 2, 4-D in the culture medium induces an embryogenic response that is associated with the increase of the endogenous levels of indole-3-acetic acid (IAA) (Michalczuk et al., 1992). The pre-treatment of plants before the induction of SE in *C. canephora* also modified the endogenous metabolism of IAA (Ayil-Gutiérrez et al., 2013).

Other PGRs, such as CKs, also participate in the development of the plants, promoting the formation of buds, delaying the aging of the leaves and, together with the auxins, stimulating cell division; both regulators are known to act synergistically (Novák and Ljung, 2017; Singh and Sinha, 2017). A high ratio between CKs and auxins stimulates the formation of shoots while that a low ratio induces the regeneration of roots and the proper establishment of meristems in *Pisum sativum* (Kotov and Kotova, 2018). These two PGR can act either synergistically or antagonistically during the induction of SE. Recent studies using synthetic reporter genes such as *DR5* for auxins and a two component system (*TCSv2*) for CKs have opened a window into the molecular mechanisms by which such interaction occurs during biosynthesis, transport and signaling (Liao et al., 2015).

In recent years there has been a significant increase in the knowledge of the signal(s) that gives rise to the SE process, but it is still unknown if auxins are the primary signal that initiates the changes in the genetic program that leads to the production of somatic embryos. In *C. canephora*, it has been shown that polar transport of the IAA is needed for the formation of the apical-basal axis (Márquez-López et al., 2018). It has also been reported that CKs are essential to maintaining basal levels of auxin biosynthesis during root and shoot development, suggesting that there is a homeostatic regulatory network to support adequate concentrations between auxins and CKs in the development of the plant (Jones et al., 2010). It is possible that a similar system is operating during the induction of SE. However, this must be tested.

## Plant Growth Regulator Response Genes During the Induction of Somatic Embryogenesis

The SE process implies the integration of endogenous signals and gene reprogramming, which unchains the signal that initiates the embryogenic process. The use of exogenous auxins, either alone or in combination with other PGRs or stress, induces the expression of different genes, which modify the genetic program of the somatic cells and regulate the transition to each of the stages during the development of SE (Loyola-Vargas and Ochoa-Alejo, 2016). Most of these genes belong to one of these four categories: transcription factors (TFs), proteins that act in the cell cycle, biosynthesis of PGR, mainly auxins, as well as proteins involved in the signaling pathway (Leljak-Levanic et al., 2015).

It is generally accepted that the SE process involves three phases: the induction of SE, the formation of the meristematic centers, and the development of the somatic embryo (Elhiti et al., 2013). Each stage comprises the interaction of multiple factors, e.g., external signals, changes in the endogenous concentrations of different PGRs, and the expression of numerous genes. Molecular studies of the induction of SE are challenging since it is difficult to identify the cells that will become new somatic embryos. However, it is possible to carry out bioinformatics analysis from transcriptomic studies gain a better picture of the candidate genes involved in the initiation of the process (Elhiti et al., 2013).

Production of the signal that leads to the changes in the genetic program requires the participation of several metabolic pathways. However, there is a consensus that auxins play a critical role in the SE process (Nic-Can and Loyola-Vargas, 2016). It is known that auxin plays a crucial role in the formation of embryo patterns in angiosperms and in gymnosperms (Larsson et al., 2008). During the induction of SE in *C. canephora*, there is an increase in the content of endogenous IAA and in the expression of the genes that code for the enzyme tryptophan aminotransferase (*TRYPTOPHAN AMINOTRANSFERASE OF ARABIDOPSIS 1*; *CcTAA1*), and for the enzyme flavin mono-oxygenase (*YUCCA*; *CcYUC1* and *CcYUC3*). Both are involved in the biosynthesis of IAA (Ayil-Gutiérrez et al., 2013).

The response of the explant is not confined to the increase in the IAA levels (Nic-Can and Loyola-Vargas, 2016). Differential gene expression can modulate the embryogenic capacity of cells, and the number of genes turned off in somatic cells to allow for the change from a somatic to an embryogenic state is higher than the number of genes that are turned on (Quiroz-Figueroa et al., 2002). In the SE of *Arabidopsis*, the modulation of several *AUXIN RESPONSE FACTORS* (*ARF*) transcripts suggests the extensive participation of auxin signaling during the process (Wójcikowska and Gaj, 2017). Almost half of the 23 ARF genes are transcribed during SE in *Arabidopsis*; six of them are upregulated and five are down-regulated. Other members of the auxin signal transduction pathway, like the putative Aux/IAA gene from *Elaeis guineensis*, *Eg*IAA9 (Ooi et al., 2012), or cotton (Yang et al., 2012), are also involved in the induction of SE. An extensive analysis of gene expression during the induction of SE in cotton shows that more than 80 genes related to the metabolism of auxins are differentially expressed (Yang et al., 2012).

## Stress and Somatic Embryogenesis

Somatic embryogenesis is a multifactorial event, which is the result of a series of physiological, biochemical and molecular changes taking place in plant cells. SE requires embryogenic competence through dedifferentiation, chromatin remodeling, programming of gene expression, and stress events mentioned above (Krishnan and Siril, 2017). In general, the SE induction includes a multitude of parallel signals that involve alterations in the levels of endogenous PGR and stress factors (Mozgová et al., 2017).

Different studies support the theory that the first stages of SE are characterized by the induction of numerous genes related to stress such as those discussed later on this review (Nic-Can et al., 2016; Nowak and Gaj, 2016). Recent evidence in potato (Kaur et al., 2018), *Pinus sylvestris* (Salo et al., 2016), *Picea asperata* (Jing et al., 2017), *Oldenlandia umbellata* (Krishnan and Siril, 2017), and *Cyathea delgadii* (Grzyb and Mikula, 2019) has revealed that the presence of different types of stress plays an essential role in the induction of SE. The main stress for cells during the induction of SE is the presence of high auxin concentration in the culture medium. Other stresses used for the induction of SE are extreme pH, heat-shock exposure or treatment with various chemical substances.

Usually, the combination of physical stress with high auxin concentration in the culture medium improves the embryogenic response. This effect was observed in *Cattleya maxim* where the effect in the SE induction was evaluated using a combination of salt (0.3 M NaCl) or osmotic stress (sorbitol 0.4 M), and the culture in a medium supplemented with 2,4-D (0.45 μM) significantly increases the percentage of protocorms with embryogenic calli (Cueva Agila et al., 2015). In some angiosperms such as *Panax ginseng*, the treatment of somatic embryos with abscisic acid (ABA) and polyethylene glycol (PEG) at a concentration of 20 μM and 3.75%, respectively, improve both the maturation and regeneration of somatic embryos compared to the untreated (Langhansová et al., 2004). However, in gymnosperms, the combined application of ABA and PEG has been shown to be necessary to stimulate the maturation and functional development of somatic embryos (Stasolla et al., 2002). For example, in *Pinus sylvestris*, embryo production is commonly induced by eliminating auxin from the culture medium, ABA addition and subsequently a PEG drying step (Salo et al., 2016). In *P. strobus*, variable amounts of water at the beginning and during the cultivation phase influences the maturation response of the embryos (Klimaszewska et al., 2000). Meanwhile, changes in water availability either by solutes or physical restriction can affect the maturation response in some conifers (Montalbán and Moncaleán, 2018). Other types of stress like heat-shock induce the SE in *Gladiolus hybridus* (Kumar et al., 2002). In cotton, several of the genes expressed during the induction of SE are related to the homeostasis of auxins and ethylene, as well as several related-stress TFs (Jin et al., 2014; Cao et al., 2017).

## Transcription Factors and Signal Transduction Involved in Somatic Embryogenesis

There is very little current information on whether the genes involved in the induction of SE work independently or in a network-like structure. However, the analysis of the interaction among different clusters of genes shows that they can act in parallel or in sequence (Ikeuchi et al., 2018). The use of transcriptomics has provided valuable. Indicates that the genes expressed during the induction of SE are divided into the categories of stress-related genes, PGR-related genes, and TFs (Cetz-Chel and Loyola-Vargas, 2016; Chu et al., 2017).

The changes in the genetic program of the cells that lead to the induction of SE require the regulation of several genes (Riechmann et al., 2000). In both angiosperms and gymnosperms, little is known about gene expression, the early stages of embryogenesis, which is crucial for the later development of the embryo (Trontin et al., 2016). For example, it has been reported that in conifers such as *Araucaria angustifolia* that the expression patterns of *AaSERK1* during SE are very similar to *SERK1* homologs of angiosperms (Steiner et al., 2012). These changes require the substantial participation of TFs. Plant genomes contain a large number (6–10%) of TFs-coding genes (Riechmann et al., 2000). Some of these TFs are shared among a variety of plant species (Supplementary Table S1). Among the TFs that have been found during the induction of SE in different species are *ABAINSENSITIVE 3 (ABI3)* (Shiota et al., 1998), *AGAMOUS LIKE (AGL)* (Harding et al., 2003; Thakare et al., 2008; Zhai et al., 2016), *BABY BOOM (BBM)* (Florez et al., 2015), *CUP SHAPED COTYLEDONS (CUC), FUSCA3 (FUS3)* (Luerûen et al., 1998), *LEAFY COTYLEDON (LEC)* (Iwase et al., 2015)*, LEAFY COTYLEDON LIKE (LIL)* (Kwong et al., 2003), *SOMATIC EMBRYOGENESIS RECEPTOR-LIKE KINASE1 (SERK1)* (Pérez-Pascual et al., 2018)*, RWP-RK DOMAIN-CONTAINING 4 (RKD4)/GROUNDED (GRD)* (Waki et al., 2011). *VIVIPAROUS1 (VP1)* (Footitt et al., 2003), and *WUSCHEL (WUS)* (Arroyo-Herrera et al., 2008; Xiao et al., 2018). In conifers, several homologs of important genes that participate during ES have been found, such as *SERK1*, *LEC1*, and *WOX2*, but it is still unknown whether they present patterns and expression functions similar to angiosperms (Trontin et al., 2016). Several of these genes are also expressed during the formation of zygotic embryos. The application of auxins or their analogs, like 2, 4-D, enhances the expression of several TFs, such as *BBM*, *WUS*, and *VP1* during the induction of SE (Awasthi et al., 2017).

In some cases, like the SE induced in wounded tissues, there is a signal that occurs before to the expression of the TFs listed in the last paragraph. The expression of *WOUND INDUCED DEDIFFERENTIATION1 (WIND1)* TF, from the AP2/ERF family, is required before the expression of *LEAFY COTYLEDON2 (LEC2)* takes place (Iwase et al., 2015). The expression of some TFs is specific to particular species; however, several others are expressed in all the systems of induction of SE studied. The roles of these TFs in the signaling process are discussed below.

### Somatic Embryogenesis Receptor Kinases (SERK)

Among the different genes that increase their expression during the induction of SE, *SERK* is the most relevant. This family of TFs is involved in a range of developmental processes that include differentiation/transdifferentiation and cellular totipotency (Pilarska et al., 2016).

The first *SERK* gene was identified in *D. carota*. It was detected in embryogenic cultures in the early days of culture in the presence of 2, 4-D. This gene is expressed in cells that develop in somatic embryos until the globular stage (Schmidt et al., 1997), just before the transition from the differentiation state to the development state. The expression of *SERK* increases several times in the embryogenic cells of *A. thaliana* (Hecht et al., 2001), *Citrus unshiu* (Shimada et al., 2005), *Dactylis glomerata* (Somleva et al., 2000), *G. hirsutum* (Pandey and Chaudhary, 2014), *Helianthus annuus* (Thomas et al., 2004), *Medicago truncatula* (Nolan et al., 2003), *Solanum tuberosum* (Sharma et al., 2008), *Vitis vinifera* (Maillot et al., 2009), *Cocos nucifera* (Pérez-Núñez et al., 2009), *Oryza sativa* (Hu et al., 2005; Ito et al., 2005), *Theobroma cacao* (de Oliveira Santos et al., 2005), *Triticum aestivum* (Singh and Khurana, 2017), *Zea mays* (Baudino et al., 2001), *Cyrtochilum loxense* (Cueva et al., 2012), and *A. angustifolia* (Steiner et al., 2012).

The evidence of the participation of *SERK* in the induction of SE has emerged from the analysis of gene expression. For example, *SERK1* is highly expressed during the formation of embryogenic cells in *in vitro* culture of *A. thaliana* and in all of the cells of the developing embryo during early SE, up until the heart stage of the somatic embryo. After this stage, the expression of *SERK1* is no longer detectable in the embryo. However, in seedlings that over-expressed *SERK1*, the mRNA exhibited a 300–400% increase in the efficiency of the initiation of SE. These results suggest that an increase in the expression levels of *SERK1* confers embryogenic competence to cells in culture (Hecht et al., 2001). In *O. sativa*, *SERK2* is expressed almost three times more in the embryogenic callus and maturation stage than in the non-embryogenic callus (Singla et al., 2009). These results suggest that different members of the *SERK* family have unique functions. Similar results have been found in *T. aestivum*. In this plant, members of the *SERK* family are expressed differentially in response to different PGR sensitivities; i.e., *SERK*2 and *SERK3* elicit auxin-specific responses while *SERK1* and *SERK5* may be mediated by the signaling pathway of brassinosteroids (Singh and Khurana, 2017).

In addition to auxins, other factors modified the expression of *SERK*. In *M. truncatula*, the expression of *SERK1* is stimulated by the presence of auxin, but not by CKs. However, when the CKs are co-administered with auxin, the level of expression of *SERK1* increases synergistically compared to the up-regulation of auxin alone. In response to a higher level of expression of *SERK*, the number of embryogenic calluses increase as well as the formation of somatic embryos (Nolan et al., 2003).

### Leafy Cotyledon (LEC)

Another important participant in the regulation of SE and plant embryo development is the *LEC* family of TFs (Guo et al., 2013). *LEC1* has an essential role in ZE and has been suggested to control diverse processes in seed development (Pelletier et al., 2017; Tvorogova and Lutova, 2018), including embryo morphogenesis, maturation phases (Guo et al., 2013), germination (Tvorogova and Lutova, 2018), and early and late embryogenesis; it also appears to allow the formation of the embryo by establishing an embryonic environment (Harada, 1999). *LEC1* is also involved in photosynthesis and chloroplast biogenesis early in seed development, and seed maturation late in the development of zygotic embryos (Pelletier et al., 2017). This gene network regulated by *LEC1* has been conserved in dicotyledonous plants that diverged tens of millions of years ago (Pelletier et al., 2017).

*LEC1* and *LEC2* were the first TFs shown to induce SE when ectopically expressed in seedlings (Stone et al., 2001). The auxin-dependent upregulation of *LEC2* has been associated with the induction of SE, whereas *LEC2* expression was markedly lower in non-embryogenic callus of *A. thaliana* (Ledwon and Gaj, 2009), suggesting that *LEC2* mediates the increase in the endogenous auxins observed during the induction of SE (Ayil-Gutiérrez et al., 2013). Similar results were found in *T. cacao*, where *LEC2* is highly expressed in the embryogenic callus and its overexpression in cotyledon explants increased the embryogenic response (Zhang et al., 2014). The ectopic overexpression of *LEC2* from *Ricinus communis* in *A. thaliana* induces the expression of TFs such as *LEC1*, *L1L*, *FUS3*, *ABI3*, and *WRINKELED1* (*WRI1*) (Kim et al., 2014). Also, the expression of the fatty acid elongase 1 (*FAE1*) and, in consequence, an accumulation of triacylglycerols, especially those containing the seed-specific fatty acid, eicosenoic acid (20:1 Δ11), in vegetative tissues was observed (Kim et al., 2014).

### WUSCHEL (WUS)

The establishment of the shoot apical meristem (SAM) is essential for SE and for shoot regeneration. These processes require the expression of *WUS*, which encodes a bifunctional homeodomain TF. *WUS* contains a highly conserved homeobox domain, and at the conserved C terminal region it has three functional domains: an acidic domain, a *WUS*-box (TLPLFPMH), and an EAR-like motif (Ikeda et al., 2009). A very important characteristic of *WUS* is its ability to move from one tissue to another. It can move from its biosynthesis site, the central zone (CZ), into the daughter cells in the peripheral zone, where it activates the transcription of *CLAVATA3* (*CVL3*), a negative regulator (Yadav et al., 2011). CLV3 moves into the extracellular space and binds to CLV1, which in turn inhibits the transcription of *WUS*. This WUS-CLV feedback system establishment maintains the stem cell pool and the development of SAM (Somssich et al., 2016; Negin et al., 2017; Zhang et al., 2017). Therefore, *WUS* has been proposed to be essential for SE (Xiao et al., 2018) and *in vitro* shoot regeneration (Zhang et al., 2017).

*WUSCHEL*, like *LEC2*, responds to the presence of auxins. Auxins trigger a signaling cascade that initiates the vegetative-to-embryogenic transition, and this transition is mediated by *WUS* (Zuo et al., 2002). The gradient of auxins that is detected during the pre-treatment of *C. canephora* plantlets and later during the initial phases of SE (Márquez-López et al., 2018) correlates with the induced *WUS* expression during SE in *A. thaliana* (Su et al., 2009).

It has been observed that *WUS*-related genes are up-regulated during SE in different species, such as *Ocotea catharinensis* Santa-Catarina et al. (2012), *M. truncatula* (Chen et al., 2009), *G. hirsutum* (Zheng et al., 2014), and *C. canephora* (Arroyo-Herrera et al., 2008). In *C. canephora*, overexpression of *WUS* enhances SE in heterologous systems (Arroyo-Herrera et al., 2008), increasing the somatic embryo production by 400%. In *G. hirsutum*, the ectopic expression of *AtWUS* promotes the proliferation and differentiation of transgenic callus and positively regulates *LEC1*, *LEC2*, and *FUS3* (Zheng et al., 2014). *WUS* overexpression enhances the induction of SE and can improve regeneration in cotton (Bouchabke-Coussa et al., 2013), and its overexpression in *A. thaliana* roots, leaf petioles, stems, or leaves induces the formation of somatic embryos (Zuo et al., 2002).

### Baby Boom (BBM)

Another key regulator of plant cell totipotency is *BBM*. *BBM* can induce embryogenesis in differentiated cells and could be a vital factor in plant embryogenesis development (Irikova et al., 2012). *BBM* triggers a set of genes like *LEC1* and *LEC2*, as well as *ABI3* and the *FUS3* network, which together activate SE (Horstman et al., 2017). The induction of SE by *BBM* is a dose-dependent mechanism and regulates the transcription of significant embryo identity genes (Horstman et al., 2017).

The *BBM* family encodes *APETALA 2/ETHYLENE RESPONSE FACTOR* (*AP2/ERF*) DNA-binding type TFs identified in the gymnosperms, angiosperms, algae, and mosses, these TFs act as a network regulation in response to biotic and abiotic stress (Kim et al., 2005). The AP2/ERF domain can bind to a GCC box, a DNA sequence involved in the ethylene response (Ohme-Takagi and Shinshi, 1995). *AP2/ERF* are divided according to the number of *AP2* domains that they contain, which are classified into subfamilies as the Dehydration-responsive 427 element-binding (DREB), *ERF*, *AP2*, and *RELATED TO ABI3/VP1* (*RAV*) genes (Gutterson and Reuber, 2004). Because *RAV* genes include another DNA-binding domain, B3, *RAV* genes are sometimes treated as a third group in the AP2/ERF family (Kim et al., 2005). The distinct feature of the BBM and BBM-like proteins is the presence of a conserved bbm-1 motif (GLSMIKTW) that is absent in other proteins of the euANT lineage (Bilichak et al., 2018). BBM activated the expression of a broad set of genes encoding proteins with potential roles in transcription, cellular signaling, cell wall biosynthesis and targeted protein turnover, such as the ACTIN DEPOLYMERIZING FACTOR9 (ADF9) (Passarinho et al., 2008).

In *A. thaliana* and *B. napus*, *BBM* changes its spatial-temporal expression in the early stages of embryogenesis (Kulinska-Lukaszek et al., 2012). Some reports show that *BBM* is expressed in the heart state of an embryo and root development (Galinha et al., 2007) and enhances the proliferation of somatic embryos (Florez et al., 2015). This response is also produced by ectopic expression of *BBM*, which changes from vegetative to embryonic growth and induces spontaneous SE in these two species (Kulinska-Lukaszek et al., 2012). The heterologous expression of *BBM* from *A. thaliana* and *B. napus* in *Nicotiana tabacum* produced an increase in the regeneration capability (Srinivasan et al., 2007). In *Capsicum annum*, both *LEC1* and *BBM* are expressed and show high levels of expression in the different phases of development of the somatic embryo (Irikova et al., 2012).

On the other hand, it is worth highlighting that *BBM* can show differential expression depending on the species and the embryogenic protocol. In a study using two species of the genus *Coffea*, it was found that while in *C. arabica* a BBM-like gene showed a twofold change in expression in embryogenic cell suspension in comparison to embryogenic calli (Silva et al., 2015), in *C. canephora* BBM1 expression was only observed after SE induction (Nic-Can et al., 2013). It has been found that the *BBM* gene is expressed at higher levels during SE in comparison to ZE in *T. cacao*, and its overexpression in *A. thaliana* and *T. cacao* led to phenotypes associated with SE that did not require exogenous hormones. However, *BBM* overexpression can inhibit the subsequent development of the somatic embryos in *T. cacao* (Florez et al., 2015), while the *BBM* overexpression in *Populus tomentosa* induced SE (Deng et al., 2009).

## Other Factors Involved in Signal Transduction During the Induction of Somatic Embryogenesis

Somatic embryogenesis signaling is a very complex process where several molecular players are involved; it would be tedious to list them all. However, there are two other major factors that need to be mentioned. One is the intervention of 14-3-3 proteins, which participate in several processes such as the development of the seeds (Zhao et al., 2015) and during the induction of SE in *Carica papaya* (Vale Ede et al., 2014). The other factor actively involved during the SE induction, process, and development is epigenetic (Us-Camas et al., 2014; De-la-Peña et al., 2015; Duarte-Aké and De-la-Peña, 2016).

### 14-3-3 Adaptor Proteins

14-3-3 adaptor proteins are a group of proteins involved in the signal transduction pathway that is shared by several PGRs involved in SE induction. These proteins are highly conserved phosphoserine-/phosphothreonine-binding proteins, discovered in the brain of mammals in 1967, with a subunit mass of 30 kDa (Carlson and Perez, 1967).

**FIGURE 2:**
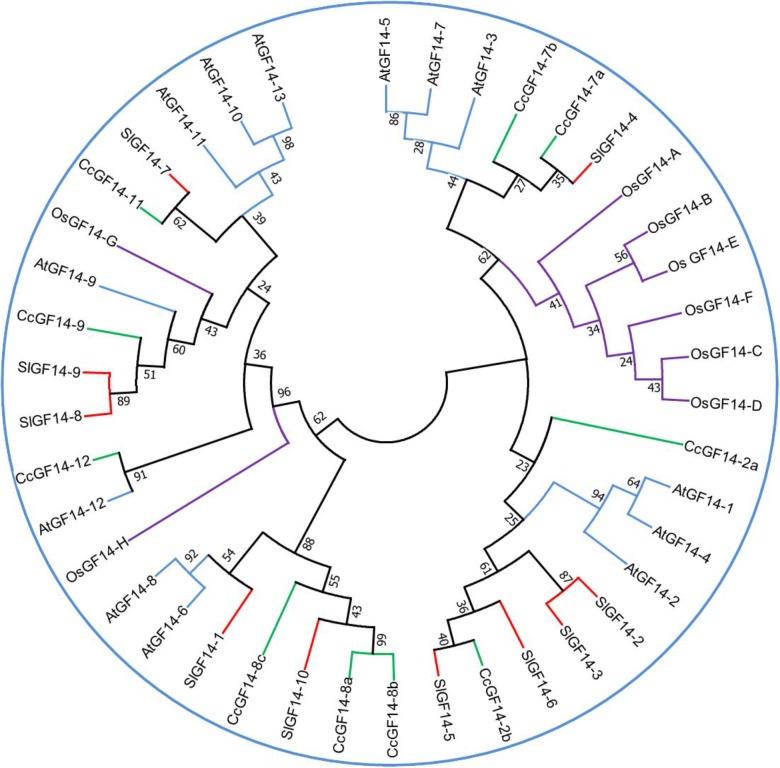
Phylogenetic tree for 14-3-3 genes family in several species. The sequences of *Coffea canephora* GF14 were obtained from http://coffee-genome.org. Rice sequences were obtained in http://rice.plantbiology.msu.edu. Tomato sequences were obtained in https://solgenomics.net/. Arabidopsis sequences were obtained in https://www.arabidopsis.org/. The sequences were aligned in the software MEGA 7 (http://www.megasoftware.net/). The percentage of replicate trees in which the associated taxa clustered together in the bootstrap test (1000 replicates) is shown next to the branches. The analysis was conducted in MEGA7 using the Neighbor-Joining method. Abbreviations: Os, *Oryza sativa*; Sl, *Solanum lycopersicum*; Cc, *Coffea canephora*; At, *Arabidopsis thaliana*.

In plants the number of members of these proteins is variable (Figure 2). There are 13 14-3-3 adaptor proteins in Arabidopsis (Rosenquist et al., 2000; DeLille et al., 2001), six in cotton (Zhang et al., 2010), 17 in tobacco (Konagaya et al., 2004), ten in tomato (Camoni et al., 2018), five in barley (Schoonheim et al., 2007), and eight in rice (Yao et al., 2007).

The use of proteomics techniques has illuminated the changes in hundreds of proteins, including the family 14-3-3, during the induction of SE (Zhao et al., 2015; Tchorbadjieva, 2016). Some 14-3-3 proteins are abundant in the embryogenic tissues of *Cyclamen persicum* (Lyngved et al., 2008), and *Larix principis* (Zhao et al., 2015). In oak, these proteins are more abundant in proliferating embryos than in mature embryos (Gomez-Garay et al., 2013).

An excellent example that shows the role of 14-3-3 proteins in the induction of SE is protein phosphatase 2A (PP2A) (Marsoni et al., 2008). This enzyme consists of a catalytic subunit and a regulatory A subunit together with a third variable B subunit (Janssens and Goris, 2001). The B subunit is the component that determines the substrate specificity and subcellular localization of PP2As. PP2A is a complex enzyme. In *A. thaliana*, there are 25 genes involved in the transcription of PP2A three subunits. The catalytic subunit (PP2Ac) is coded by five genes, three other genes encoding A subunits and seventeen different genes encoding B subunits (Farkas et al., 2007). The subunit A is essential for auxin transport (Michniewicz et al., 2007), while the 65 kDa regulatory subunit of PP2A has regulatory functions. The subunit A has been associated with the SE process (Marsoni et al., 2008). There is a noticeable increase in phosphorylation of specific proteins in embryogenic cultures compared to the non-embryogenic cells of *C. persicum*, which has been correlated with higher levels of PP2A and a 14-3-3-like protein (Lyngved et al., 2008). Other components of the signal transduction cascade, such as G proteins and calreticulin, increased during cyclamen SE (Rensing et al., 2005). It has been suggested that the increase in the regulatory subunit of PP2A and 14-3-3 proteins during the induction of SE is related to the stress conditions produced by the *in vitro* culturing of *C. persicum* (Lyngved et al., 2008) and *L. principis* embryogenic cultures (Zhao et al., 2015).

## Epigenetics

In recent years, epigenetic mechanisms during chromatin remodeling have emerged as critical factors in SE. Epigenetic modifications are an essential part of the signaling pathway that leads to changes in the genetic program of the cells and the development of somatic embryos. There is evidence that shows that changes in the chromatin are able to control totipotency in plant cells (Duarte-Aké and De-la-Peña, 2016; Kumar and van Staden, 2017). The level to which chromatin reprogramming is required before SE induction depends on several factors, such as origin of the explant, the culture medium, the genetic background of the mother plant, and especially the amount of PGR used (De-la-Peña et al., 2015).

DNA methylation is important for somatic embryo development (Nic-Can et al., 2013; Yakovlev et al., 2016). In general, higher global DNA methylation has been found in non-embryogenic cultures of *Pinus radiata* (Bravo et al., 2017), *P. nigra* (Noceda et al., 2009), *Rosa* x *hybrid* (Xu et al., 2004), and *Eleutherococcus senticosus* (Chakrabarty et al., 2003), while low global DNA methylation has been found in embryogenic cultures of several plants. In *Quercus alba* DNA is demethylation during the induction of SE (Corredoira et al., 2017), as well as during the generation of pro-embryogenic mass, but it gradually increases as the embryo is developing (LoSchiavo et al., 1989). Similar results were observed during the SE of *C. canephora* (Nic-Can et al., 2013), where the proembryogenic mass had lower DNA methylation, while the maturation of the embryos was marked by a gradual increase in the global levels of methylation.

In *A. thaliana* it was found that both *de novo* DNA methylation and maintenance of it are required for the regulation of SE (Grzybkowska et al., 2018), and similar results were found in *Picea abies* (Yakovlev et al., 2016). Changes in the global DNA methylation pattern during long-term subcultures could lead to the loss of the embryonic potential of proembryogenic masses (Fraga et al., 2016).

In order to prove that in fact DNA methylation is strongly related to SE, pharmacological experiments have been conducted in several plant species. The application of 5-azacitidine (5-AzaC; a demethylating agent) decreased the levels of global DNA methylation in *A. thaliana* and inhibited the induction of SE (Grzybkowska et al., 2018). Similar results have been found in *M. truncatula* (Santos and Fevereiro, 2002), *D. carota* (Yamamoto et al., 2005), and *C. canephora* (Nic-Can et al., 2013). Furthermore, *LEC1*, *LEC2*, and *BBM* genes were up-regulated in the *drm1drm2* and *drm1drm2cmt3* mutants, an upregulation that was related to an improvement in the SE response (Grzybkowska et al., 2018). In *T. cacao*, DNA methylation increased during the induction of SE, and treatment with 5-AzaC led to the recovery of SE potential in aged cultures (Pila Quinga et al., 2017). 5-AzaC is not the only drug used to disrupt epigenetic modifications; trichostatin A (TSA), the function of which is inhibiting histone deacetylases (HDACs), has a positive effect on gene expression. The inhibition of HDACs has also led to an increase in the number of haploid embryos produced by heat stress in *B. napus* (Li et al., 2014). In fact, the treatment with TSA of germinating spruce somatic embryos preserves their embryogenic nature (Uddenberg et al., 2011). In the double mutant hda6/hda19, the upregulation of *LEC1*, *FUS3*, and *ABI3* genes was evident in germinating Arabidopsis seeds (Tanaka et al., 2008). These double mutants also led to the production of somatic embryos in the leaves of Arabidopsis (Tanaka et al., 2008).

Histones’ posttranslational modifications have been implicated in the formation of somatic embryos. Histone deacetylation may also play a role in the reprogramming of cells in the early stages of SE (De-la-Peña et al., 2015; Lee et al., 2016), since the levels of histone acetylation and the activity of HDACs change in response to the presence of exogenous PGR during the induction of SE.

There are several tissue-specific events involving H3K27me3. The loss of this mark upregulates the auxin pathway and its increase leads to the repression of leaf identity (He et al., 2012). Polycomb repressive complex 2 (PRC2) is involved in the methylation of lysine 27 in histone H3 (Molitor et al., 2014). Double mutants of the *PRC2* gene, which functions as a histone methyltransferase, *CLF* and *SWN* or *VERNALIZATION 2* (*VRN2*) and *EMBRYONIC FLOWER2* (*EMF2*) form callus on the shoot apex, lead to indirect somatic embryo formation and ectopic roots (Chanvivattana et al., 2004). A *PRC2* mutant root hairs fail to maintain their differentiated state and form unorganized cell masses and eventually somatic embryos from callus (Ikeuchi et al., 2015). The effect of silencing genes of the PCR2 family in inducing SE depends on the explant. In tissues where *PCR2* is scarcely active, the production of somatic embryos is efficient; however, in the tissues where it is highly expressed somatic embryos do not form (Liu et al., 2016; Mozgová et al., 2017).

## Concluding Remarks

Since the 1950s, the research on the SE process has gone from empirical approaches to a more methodical investigation leading to the production of somatic embryos (Loyola-Vargas, 2016). We are well on the way to understanding the role of auxins and other PGRs, as well as stress, on the induction of SE (Nic-Can et al., 2016; Nic-Can and Loyola-Vargas, 2016). We now have a set of genes that, in some cases, can be used as markers of the initiation of SE. However, the signal pathway from the initial signal to the first steps of the development of the somatic embryo remains practically unknown.

**FIGURE 3:**
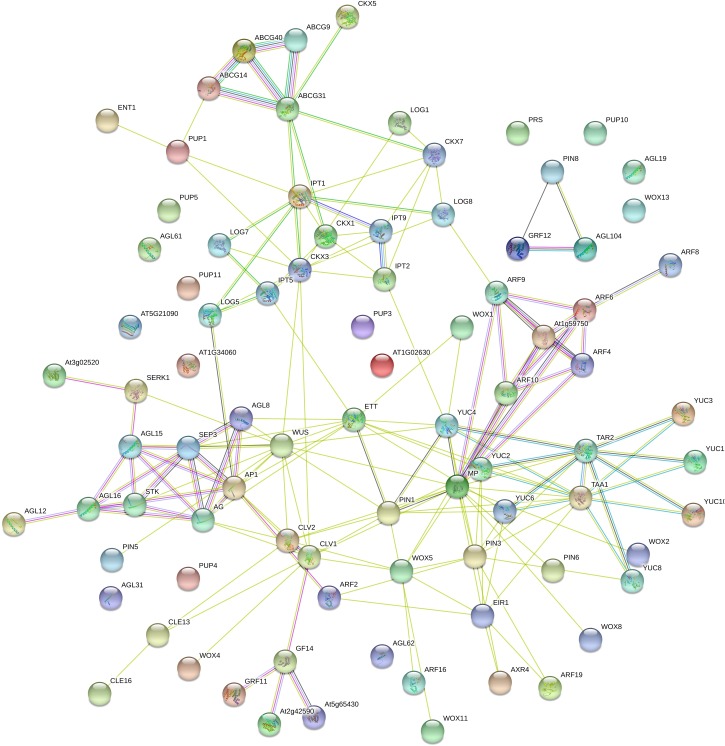
Interactome of *Coffea canephora* proteins related to somatic embryogenesis. *C. canephora* proteins were compared with *Arabidopsis thaliana* proteins using STRING software (https://string-db.org/); max score and sequence coverage were the principal parameters in the identification and selection. Colored lines mean the following: Gene neighborhood (dark green), co-expression (black), experimentally determined (pink), Text-mining (light green), from a curated database (light blue), protein homology (gray), and gene co-occurrence (dark blue). The description of the roles of every gene in the interactome is listed in Supplementary Table S2.

Scientists have just begun to understand the complex network of interactions among a set of TFs, the endogenous concentrations of auxins, CKs, ABA, ethylene and salicylic acid, their transport and receptors, and the origin of the explant that lead to the establishment of a somatic embryo (Figure 3 and Supplementary Table S2).

Current scientific knowledge lets us hypothesize that the initial signal, stress or the signals produced by the PGRs induce a change in the endogenous concentration of several PGRs, especially auxins and CKs. The differences in the relationship between the auxins and CKs lead to the expression of TFs and ARF, which in turn modify the cell wall, a vital component in the cell differentiation process. Once the cell(s) are settled into the SE pathway, the expression of TFs, such as *BBM*, *SERC*, and *LEC*, leads to downstream changes in the endogenous content of different compounds and produces a cascade of events, such as chromatin remolding, that drives the induction of SE. However, there are still many questions to answer to understand how the life of a somatic embryo begins. The roles of ethylene, salicylic acid, the organization of the cytoskeleton, brassinosteroids, and other compounds remain to be elucidated.

The overexpression of genes such as *WUS*, *BBM*, and *LEC* has been used to induce SE in different plant species. This approach has been instrumental in understanding the role of different genes during the induction of SE; however, under certain conditions, the overexpression also inhibits the induction of SE. This means that under the present state of the art, every gene and every plant species must be tested, before all of the pieces of the puzzle are in place.

Increasing knowledge of the induction of SE and of the development of somatic embryos will lead to the development of multiple biotechnological applications and new opportunities for the understanding of the fundamental aspects of SE. In particular, the alteration in the methylation or acetylation profile of DNA and/or histones by genome-editing techniques holds great promise to increase the production and to improve the quality of crops of agronomical importance (Karim et al., 2016).

## Author Contributions

VL-V developed the idea. All authors drafted the manuscript.

## Conflict of Interest Statement

The authors declare that the research was conducted in the absence of any commercial or financial relationships that could be construed as a potential conflict of interest.

## References

[B1] Abohatem M. A. Bakil Y. Baaziz M. (2017). “Plant regeneration from somatic embryogenic suspension cultures of date palm,” in *Date Palm Biotechnology Protocols*, Vol. I, eds Al-Khayri J. Jain S. Johnson D. (New York, NY: Springer), 203–214. 10.1007/978-1-4939-7156-5_17 28755347

[B2] Arroyo-Herrera A. Ku-Gonzalez A. Canche-Moo R. Quiroz-Figueroa F. R. Loyola-Vargas V. M. Rodriguez-Zapata L. C. (2008). Expression of *WUSCHEL* in *Coffea canephora* causes ectopic morphogenesis and increases somatic embryogenesis. *Plant Cell Tissue Organ Cult.* 94 171–180. 10.1007/s11240-008-9401-1

[B3] Awasthi P. Sharma V. Kaur N. Kaur N. Pandey P. Tiwari S. (2017). Genome-wide analysis of transcription factors during somatic embryogenesis in banana (*Musa* spp.) cv. Grand naine. *PLoS One* 12:e0182242. 10.1371/journal.pone.0182242 28797040 PMC5552287

[B4] Ayil-Gutiérrez B. A. Galaz-Ávalos R. M. Peña-Cabrera E. Loyola-Vargas V. M. (2013). Dynamics of the concentration of IAA and some of its conjugates during the induction of somatic embryogenesis in *Coffea canephora*. *Plant Signal. Behav.* 8:e26998. 10.4161/psb.26998 24299659 PMC4091420

[B5] Baudino S. Hansen S. Brettschneider R. Hecht V. F. G. Dresselhaus T. Lörz H. (2001). Molecular characterisation of two novel maize LRR receptor-like kinases, which belong to the *SERK* gene family. *Planta* 213 1–10. 10.1007/s004250000471 11523644

[B6] Bilichak A. Luu J. Jiang F. Eudes F. (2018). Identification of BABY BOOM homolog in bread wheat. *Agric. Gene* 7 43–51. 10.1016/j.aggene.2017.11.002

[B7] Bouchabke-Coussa O. Obellianne M. Linderme D. Montes E. Maia-Grondard A. Vilaine F. (2013). Wuschel overexpression promotes somatic embryogenesis and induces organogenesis in cotton (*Gossypium hirsutum* L.) tissues cultured in vitro. *Plant Cell Rep.* 32 675–686. 10.1007/s00299-013-1402-9 23543366

[B8] Bravo S. Bertín A. Turner A. Sepúlveda F. Jopia P. Parra M. A. J. (2017). Differences in DNA methylation, DNA structure and embryogenesis-related gene expression between embryogenic and non embryogenic lines of *Pinus radiata* D. don. *Plant Cell Tissue Organ Cult.* 130 521–529. 10.1007/s11240-017-1242-3

[B9] Camoni L. Visconti S. Aducci P. Marra M. (2018). 14-3-3 Proteins in plant hormone signaling: doing several things at once. *Front. Plant Sci.* 9:297. 10.3389/fpls.2018.00297 29593761 PMC5859350

[B10] Cao A. Zheng Y. Yu Y. Wang X. Shao D. Sun J. (2017). Comparative transcriptome analysis of SE initial dedifferentiation in cotton of different SE capability. *Sci. Rep.* 7:8583. 10.1038/s41598-017-08763-8 28819177 PMC5561258

[B11] Carlson F. D. Perez V. J. (1967). “Specific acidic proteins of the nervous system,” in *Physiological and Biochemical Aspects of Nervous Integration*, ed. Carlson F. D. (Cliffs, NJ: Prentice-Hall), 343–359.

[B12] Cetz-Chel J. E. Loyola-Vargas V. M. (2016). “Transcriptome profile of somatic embryogenesis,” in *Somatic Embryogenesis. Fundamental Aspects and Applications*, eds Loyola-Vargas V. M. Ochoa-Alejo N. (Cham: Springer), 39–52. 10.1007/978-3-319-33705-0_4

[B13] Chakrabarty D. Yu K. W. Paek K. Y. (2003). Detection of DNA methylation changes during somatic embryogenesis of Siberian ginseng (*Eleutherococcus senticosus*). *Plant Sci.* 165 61–68. 10.1016/S0168-9452(03)00127-4

[B14] Chanvivattana Y. Bishopp A. Schubert D. Stock C. Moon Y. H. Sung Z. R. (2004). Interaction of polycomb-group proteins controlling flowering in *Arabidopsis*. *Development* 131 5263–5276. 10.1242/dev.01400 15456723

[B15] Chen S. Kurdyukov S. Kereszt A. Wang X. Gresshoff P. Rose R. (2009). The association of hoemeobox gene expression with stem cell formation and morphogenesis in cultured *Medicago truncatula*. *Planta* 230 827–840. 10.1007/s00425-009-0988-1 19639337 PMC2729979

[B16] Chu Z. Chen J. Sun J. Dong Z. Yang X. Wang Y. (2017). *De novo* assembly and comparative analysis of the transcriptome of embryogenic callus formation in bread wheat (*Triticum aestivum* L.). *BMC Plant Biol.* 17:244. 10.1186/s12870-017-1204-2 29258440 PMC5735865

[B17] Corredoira E. Cano V. Bárány I. Solís M. T. Rodríguez H. Vieitez A. M. (2017). Initiation of leaf somatic embryogenesis involves high pectin esterification, auxin accumulation and DNA demethylation in *Quercus alba*. *J. Plant Physiol.* 213 42–54. 10.1016/j.jplph.2017.02.012 28315794

[B18] Cueva A. Concia L. Cella R. (2012). Molecular characterization of a *Cyrtochilum loxense* somatic embryogenesis Receptor-like Kinase (SERK) gene expressed during somatic embryogenesis. *Plant Cell Rep.* 31 1129–1139. 10.1007/s00299-012-1236-x 22350407

[B19] Cueva Agila A. Y. Guachizaca I. Cella R. (2015). Combination of 2, 4-D and stress improves indirect somatic embryogenesis in *Cattleya maxima* Lindl. *Plant Biosyst.* 149 235–241. 10.1080/11263504.2013.797033

[B20] de Oliveira Santos M. Romano E. Yotoko K. S. C. Tinoco M. L. P. Andrade Diaz B. B. Aragao F. J. L. (2005). Characterisation of the cacao somatic embryogenesis receptor-like kinase (*SERK*) gene expressed during somatic embryogenesis. *Plant Sci.* 168 723–729. 10.1016/j.plantsci.2004.10.004

[B21] De-la-Peña C. Nic-Can G. I. Galaz-Ávalos R. M. Avilez-Montalvo R. N. Loyola-Vargas V. M. (2015). The role of chromatin modifications in somatic embryogenesis in plants. *Front. Plant Sci.* 6:635. 10.3389/fpls.2015.00635 26347757 PMC4539545

[B22] DeLille J. M. Sehnke P. C. Ferl R. J. (2001). The Arabidopsis 14-3-3 family of signaling regulators. *Plant Physiol.* 126 35–38. 10.1104/pp.126.1.35 11351068 PMC1540106

[B23] Deng W. Luo K. Li Z. Yang Y. (2009). A novel method for induction of plant regeneration via somatic embryogenesis. *Plant Sci.* 177 43–48. 10.1016/j.plantsci.2009.03.009

[B24] Dodeman V. L. Ducreux G. Kreis M. (1997). Zygotic embryogenesis *versus* somatic embryogenesis. *J. Exp. Bot.* 48 1493–1509. 10.1093/jxb/48.8.1493

[B25] Duarte-Aké F. De-la-Peña C. (2016). “Epigenetic advances in somatic embryogenesis in sequenced genome crops,” in *Somatic Embryogenesis: Fundamental Aspects and Applications*, eds Loyola-Vargas V. M. Ochoa-Alejo N. (Cham: Springer International Publishing), 81–102. 10.1007/978-3-319-33705-0_6

[B26] Elhiti M. Stasolla C. Wang A. (2013). Molecular regulation of plant somatic embryogenesis. *In Vitro Cell. Dev. Biol. Plant* 49 631–642. 10.1007/s11627-013-9547-3

[B27] Farkas I. Dombradi V. Miskei M. Szabados L. Koncz C. (2007). *Arabidopsis* PPP family of serine/threonine phosphatases. *Trends Plant Sci.* 12 169–176. 10.1016/j.tplants.2007.03.003 17368080

[B28] Florez S. L. Erwin R. L. Maximova S. N. Guiltinan M. J. Curtis W. R. (2015). Enhanced somatic embryogenesis in *Theobroma cacao* using the homologous BABY BOOM transcription factor. *BMC Plant Biol.* 15:121. 10.1186/s12870-015-0479-4 25976599 PMC4449528

[B29] Footitt S. Ingouff M. Clapham D. von Arnold S. (2003). Expression of the viviparous 1 (Pavp1) and p34cdc2 protein kinase (cdc2Pa) genes during somatic embryogenesis in Norway spruce (*Picea abies* [L.] Karst). *J. Exp. Bot.* 54 1711–1719. 10.1093/jxb/erg178 12754264

[B30] Fraga H. P. F. Vieira L. N. Heringer A. S. Puttkammer C. C. Silveira V. Guerra M. P. (2016). DNA methylation and proteome profiles of *Araucaria angustifolia* (Bertol.) Kuntze embryogenic cultures as affected by plant growth regulators supplementation. *Plant Cell Tissue Organ Cult.* 125 353–374. 10.1007/s11240-016-0956-y

[B31] Fuentes-Cerda C. F. J. Monforte-González M. Méndez-Zeel M. Rojas-Herrera R. Loyola-Vargas V. M. (2001). Modification of the embryogenic response of *Coffea arabica* by nitrogen source. *Biotechnol. Lett.* 23 1341–1343. 10.1023/A:1010545818671

[B32] Galinha C. Hofhuis H. Luijten M. Willemsen V. Blilou I. Heidstra R. (2007). PLETHORA proteins as dose-dependent master regulators of *Arabidopsis* root development. *Nature* 449 1053–1057. 10.1038/nature06206 17960244

[B33] Garcês H. M. P. Sinha N. (2009). The ‘mother of thousands’ (*Kalanchoë daigremontiana*): a plant model for asexual reproduction and CAM studies. *Cold Spring Harb. Protoc.* 2009:db.emo133. 10.1101/pdb.emo133 20147034

[B34] Goldberg R. B. De Paiva G. Yadegari R. (1994). Plant embryogenesis: zygote to seed. *Science* 266 605–614. 10.1126/science.266.5185.605 17793455

[B35] Gomez-Garay A. Lopez J. A. Camafeita E. Bueno M. A. Pintos B. (2013). Proteomic perspective of *Quercus suber* somatic embryogenesis. *J. Proteom.* 93 314–325. 10.1016/j.jprot.2013.06.006 23770300

[B36] Grzyb M. Kalandyk A. Mikula A. (2018). Effect of TIBA, fluridone and salicylic acid on somatic embryogenesis and endogenous hormone and sugar contents in the tree fern *Cyathea delgadii* Sternb. *Acta Physiol. Plant.* 40:1 10.1007/s11738-017-2577-4

[B37] Grzyb M. Mikula A. (2019). Explant type and stress treatment determine the uni- and multicellular origin of somatic embryos in the tree fern *Cyathea delgadii* Sternb. *Plant Cell Tissue Organ Cult.* 136 221–230. 10.1007/s11240-018-1507-5

[B38] Grzybkowska D. Morosczyk J. Wójcikowska B. Gaj M. D. (2018). Azacitidine (5-AzaC)-treatment and mutations in DNA methylase genes affect embryogenic response and expression of the genes that are involved in somatic embryogenesis in Arabidopsis. *Plant Growth Regul.* 85 243–256. 10.1007/s10725-018-0389-1

[B39] Guo F. Liu C. Xia H. Bi Y. Zhao C. Zhao S. (2013). Induced expression of AtLEC1 and AtLEC2 differentially promotes somatic embryogenesis in transgenic tobacco plants. *PLoS One* 8:e71714. 10.1371/journal.pone.0071714 23951228 PMC3741171

[B40] Gutterson N. Reuber T. L. (2004). Regulation of disease resistance pathways by AP2/ERF transcription factors. *Curr. Opin. Plant Biol.* 7 465–471. 10.1016/j.pbi.2004.04.007 15231271

[B41] Harada J. J. (1999). Signaling in plant embryogenesis. *Curr. Opin. Plant Biol.* 2 23–27. 10.1016/S1369-5266(99)80005-3 10047570

[B42] Harada J. J. Kwong R. W. (eds). (2002). “Plant embryogenesis (zygotic and somatic),” in *Encyclopedia of Life Sciences*, (Hoboken, NJ: John Wiley and Sons), 1–7. 10.1002/9780470015902.a0002042.p

[B43] Harding E. W. Tang W. Nichols K. W. Fernandez D. E. Perry S. E. (2003). Expression and maintenance of embryogenic potential is enhanced through constitutive expression of AGAMOUS-like 15. *Plant Physiol.* 133 653–663. 10.1104/pp.103.023499 14512519 PMC219041

[B44] He C. Chen X. Huang H. Xu L. (2012). Reprogramming of H3K27me3 is critical for acquisition of pluripotency from cultured *Arabidopsis* tissues. *PLoS Genet.* 8:e1002911. 10.1371/journal.pgen.1002911 22927830 PMC3426549

[B45] Hecht V. Vielle-Calzada J. P. Hartog M. V. Schmidt E. D. L. Boutilier K. Grossniklaus U. (2001). The Arabidopsis *SOMATIC EMBRYOGENESIS RECEPTOR KINASE 1* gene is expressed in developing ovules and embryos and enhances embryogenic competence in culture. *Plant Physiol.* 127 803–816. 10.1104/pp.010324 11706164 PMC129253

[B46] Horstman A. Li M. Heidmann I. Weemen M. Chen B. Muiño J. M. (2017). The BABY BOOM transcription factor activates the LEC1-ABI3-FUS3-LEC2 network to induce somatic embryogenesis. *Plant Physiol.* 175 848–857. 10.1104/pp.17.00232 28830937 PMC5619889

[B47] Hu H. Xiong L. Yang Y. (2005). Rice *SERK1* gene positively regulates somatic embryogenesis of cultured cell and host defense response against fungal infection. *Planta* 222 107–117. 10.1007/s00425-005-1534-4 15968510

[B48] Ikeda M. Mitsuda N. Ohme-Takagi M. (2009). Arabidopsis WUSCHEL is a bifunctional transcription factor that acts as a repressor in stem cell regulation and as an activator in floral patterning. *Plant Cell* 21 3493–3505. 10.1105/tpc.109.069997 19897670 PMC2798335

[B49] Ikeuchi M. Iwase A. Rymen B. Harashima H. Shibata M. Ohnuma M. (2015). PRC2 represses dedifferentiation of mature somatic cells in *Arabidopsis*. *Nat. Plants* 1:15089. 10.1038/nplants.2015.89 27250255

[B50] Ikeuchi M. Shibata M. Rymen B. Iwase A. Bågman A. M. Watt L. (2018). A gene regulatory network for cellular reprogramming in plant regeneration. *Plant Cell Physiol.* 59 770–782. 10.1093/pcp/pcy013 29462363 PMC6018650

[B51] Irikova T. Grozeva S. Denev I. (2012). Identification of *BABY BOOM* and *LEAFY COTYLEDON* genes in sweet pepper (*Capsicum annuum* L.) genome by their partial gene sequences. *Plant Growth Regul.* 67 191–198. 10.1007/s10725-012-9676-4

[B52] Ito Y. Takaya K. Kurata N. (2005). Expression of SERK family receptor-like protein kinase genes in rice. *Biochim. Biophys. Acta* 1730 253–258. 10.1016/j.bbaexp.2005.06.007 16081169

[B53] Iwase A. Mita K. Nonaka S. Ikeuchi M. Koizuka C. Ohnuma M. (2015). WIND1-based acquisition of regeneration competency in Arabidopsis and rapeseed. *J. Plant Res.* 128 389–397. 10.1007/s10265-015-0714-y 25810222

[B54] Janssens V. Goris J. (2001). Protein phosphatase 2A: a highly regulated family of serine/threonine phosphatases implicated in cell growth and signalling. *Biochem. J.* 353 417–439. 10.1042/bj3530417 11171037 PMC1221586

[B55] Jin F. Hu L. Yuan D. Xu J. Gao W. He L. (2014). Comparative transcriptome analysis between somatic embryos (SEs) and zygotic embryos in cotton: evidence for stress response functions in SE development. *Plant Biotechnol. J.* 12 161–173. 10.1111/pbi.12123 24112122

[B56] Jing D. Zhang J. Xia Y. Kong L. OuYang F. Zhang S. (2017). Proteomic analysis of stress-related proteins and metabolic pathways in *Picea asperata* somatic embryos during partial desiccation. *Plant Biotechnol. J.* 15 27–38. 10.1111/pbi.12588 27271942 PMC5253475

[B57] Jones B. Gunneras S. A. Petersson S. V. Tarkowski P. Graham N. May S. (2010). Cytokinin regulation of auxin synthesis in *Arabidopsis* involves a homeostatic feedback loop regulated via auxin and cytokinin signal transduction. *Plant Cell* 22 2956–2969. 10.1105/tpc.110.074856 20823193 PMC2965550

[B58] Kamada H. Harada H. (1979). Studies on the organogenesis in carrot tissue cultures I. Effects of growth regulators on somatic embryogenesis and root formation. *Z. Pflanzenphysiol.* 91 255–266. 10.1016/S0044-328X(79)80099-9

[B59] Karim R. Nuruzzaman M. Khalid N. Harikrishna J. A. (2016). Importance of DNA and histone methylation in *in vitro* plant propagation for crop improvement: a review. *Ann. Appl. Biol.* 169 1–16. 10.1111/aab.12280

[B60] Kaur A. Reddy M. S. Kumar A. (2018). Direct somatic embryogenesis of potato [*Solanum tuberosum* (L.)] cultivar “Kufri Chipsona 2”. *Plant Cell Tissue Organ Cult.* 134 457–466. 10.1007/s11240-018-1435-4

[B61] Kim H. U. Jung S. J. Lee K. R. Kim E. H. Lee S. M. Roh K. H. (2014). Ectopic overexpression of castor bean LEAFY COTYLEDON2 (LEC2) in Arabidopsis triggers the expression of genes that encode regulators of seed maturation and oil body proteins in vegetative tissues. *FEBS Open Bio* 4 25–32. 10.1016/j.fob.2013.11.003 24363987 PMC3863707

[B62] Kim S. Soltis P. S. Wall K. Soltis D. E. (2005). Phylogeny and domain evolution in the APETALA2-like gene family. *Mol. Biol. Evol.* 23 107–120. 10.1093/molbev/msj014 16151182

[B63] Klimaszewska K. Bernier-Cardou M. Cyr D. R. Sutton B. C. S. (2000). Influence of gelling agents on culture medium gel strength, water availability, tissue water potential, and maturation response in embryogenic cultures of *Pinus strobus* L. *In Vitro Cell. Dev. Biol. Plant* 36 279–286. 10.1007/s11627-000-0051-1

[B64] Konagaya K. I. Matsushita Y. Kasahara M. Nyunoya H. (2004). Members of 14-3-3 protein isoforms interacting with the resistance gene product N and the elicitor of *Tobacco mosaic virus*. *J. Gen. Plant Pathol.* 70 221–231. 10.1007/s10327-003-0113-4

[B65] Kotov A. A. Kotova L. M. (2018). Auxin-cytokinin interactions in regulating correlative inhibition in two-branched pea seedlings. *J. Exp. Bot.* 69 2967–2978. 10.1093/jxb/ery117 29590457 PMC5972627

[B66] Krishnan S. R. S. Siril E. A. (2017). Auxin and nutritional stress coupled somatic embryogenesis in *Oldenlandia umbellata* L. *Physiol. Mol. Biol. Plants* 23 471–475. 10.1007/s12298-017-0425-z 28461734 PMC5391357

[B67] Kulinska-Lukaszek K. Tobojka M. Adamiok A. Kurczynska E. (2012). Expression of the *BBM* gene during somatic embryogenesis of *Arabidopsis thaliana*. *Biol. Plant.* 56 389–394. 10.1007/s10535-012-0105-3

[B68] Kumar A. Palni L. M. Sood A. Sharma M. Palni U. T. Gupta A. K. (2002). Heat-shock induced somatic embryogenesis in callus cultures of gladiolus in the presence of high sucrose. *J. Hortic. Sci. Biotechnol.* 77 73–78. 10.1080/14620316.2002.11511460

[B69] Kumar V. van Staden J. (2017). New insights into plant somatic embryogenesis: an epigenetic view. *Acta Physiol. Plant.* 39:194 10.1007/s11738-017-2487-5

[B70] Kwong R. W. Bui A. Q. Lee H. Kwong L. W. Fischer R. L. Goldberg R. B. (2003). LEAFY COTYLEDON1-LIKE defines a class of regulators essential for embryo development. *Plant Cell* 15 5–18. 10.1105/tpc.006973 12509518 PMC143447

[B71] Langhansová L. Konradová H. Vanek T. (2004). Polyethylene glycol and abscisic acid improve maturation and regeneration of *Panax ginseng* somatic embryos. *Plant Cell Rep.* 22 725–730. 10.1007/s00299-003-0750-2 14735313

[B72] Larsson E. Sitbon F. Ljung K. von Arnold S. (2008). Inhibited polar auxin transport results in aberrant embryo development in Norway spruce. *New Phytol.* 177 356–366. 10.1111/j.1469-8137.2007.02289.x 18042199

[B73] Ledwon A. Gaj M. (2009). *LEAFY COTYLEDON2* gene expression and auxin treatment in relation to embryogenic capacity of *Arabidopsis* somatic cells. *Plant Cell Rep.* 28 1677–1688. 10.1007/s00299-009-0767-2 19763577

[B74] Lee K. Park O. S. Jung S. J. Seo P. J. (2016). Histone deacetylation-mediated cellular dedifferentiation in *Arabidopsis*. *J. Plant Physiol.* 191 95–100. 10.1016/j.jplph.2015.12.006 26724747

[B75] Leljak-Levanic D. Mihaljevic S. Bauer N. (2015). Somatic and zygotic embryos share common developmental features at the onset of plant embryogenesis. *Acta Physiol. Plant.* 37 1–14. 10.1007/s11738-015-1875-y

[B76] Li H. Soriano M. Cordewener J. Muiño J. M. Riksen T. Fukuoka H. (2014). The histone deacetylase inhibitor trichostatin A promotes totipotency in the male gametophyte. *Plant Cell* 26 195–209. 10.1105/tpc.113.116491 24464291 PMC3963568

[B77] Liao C. Y. Smet W. Brunoud G. Yoshida S. Vernoux T. Weijers D. (2015). Reporters for sensitive and quantitative measurement of auxin response. *Nat. Meth.* 12 207–210. 10.1038/nmeth.3279 25643149 PMC4344836

[B78] Liu J. Deng S. Wang H. Ye J. Wu H. W. Sun H. X. (2016). *CURLY LEAF* regulates gene sets coordinating seed size and lipid biosynthesis in Arabidopsis. *Plant Physiol.* 171 424–436. 10.1104/pp.15.01335 26945048 PMC4854673

[B79] LoSchiavo F. Pitto L. Giuliano G. Torti G. Nuti-Ronchi V. Marazziti D. (1989). DNA methylation of embryogenic carrot cell cultures and its variations as caused by mutation, differentiation, hormones and hypomethylating drugs. *Theor. Appl. Genet.* 77 325–331. 10.1007/BF00305823 24232608

[B80] Loyola-Vargas V. M. (2016). “The history of somatic embryogenesis,” in *Somatic Embryogenesis. Fundamental Aspects and Applications*, eds Loyola-Vargas V. M. Ochoa-Alejo N. (Cham: Springer), 11–22. 10.1007/978-3-319-33705-0_2

[B81] Loyola-Vargas V. M. Ochoa-Alejo N. (2016). “Somatic embryogenesis. An overview,” in *Somatic Embryogenesis. Fundamental Aspects and Applications*, eds Loyola-Vargas V. M. Ochoa-Alejo N. (Cham: Springer), 1–10. 10.1007/978-3-319-33705-0_1

[B82] Luerûen H. Kirik V. Herrmann P. Miséra S. (1998). *FUSCA3*encodes a protein with a conserved VP1/ABI3-like B3 domain which is of functional importance for the regulation of seed maturation in *Arabidopsis thaliana*. *Plant J.* 15 755–764. 10.1046/j.1365-313X.1998.00259.x 9807814

[B83] Lyngved R. Renaut J. Hausman J. F. Iversen T. H. Hvoslef-Eide A. K. (2008). Embryo-specific proteins in *Cyclamen persicum* analyzed with 2-D DIGE. *J. Plant Growth Regul.* 27:353 10.1007/s00344-008-9061-8

[B84] Maillot P. Lebel S. Schellenbaum P. Jacques A. Walter B. (2009). Differential regulation of SERK, LEC1-Like and pathogenesis-related genes during indirect secondary somatic embryogenesis in grapevine. *Plant Physiol. Biochem.* 47 743–752. 10.1016/j.plaphy.2009.03.016 19406655

[B85] Márquez-López R. E. Pérez-Hernández C. A. Kú-González Á. Galaz-Ávalos R. M. Loyola-Vargas V. M. (2018). Localization and transport of indole-3-acetic acid during somatic embryogenesis in *Coffea canephora*. *Protoplasma* 255 695–708. 10.1007/s00709-017-1181-1 29119309

[B86] Marsoni M. Bracale M. Espen L. Prinsi B. Negri A. Vannini C. (2008). Proteomic analysis of somatic embryogenesis in *Vitis vinifera*. *Plant Cell Rep.* 27 347–356. 10.1007/s00299-007-0438-0 17874111

[B87] Michalczuk L. Cooke T. J. Cohen J. D. (1992). Auxin levels at different stages of carrot somatic embryogenesis. *Phytochemistry* 31 1097–1103. 10.1016/0031-9422(92)80241-6

[B88] Michniewicz M. Zago M. K. Abas L. Weijers D. Schweighofer A. Meskiene I. (2007). Antagonistic regulation of PIN phosphorylation by PP2A and PINOID directs auxin flux. *Cell* 130 1044–1056. 10.1016/j.cell.2007.07.033 17889649

[B89] Molitor A. M. Bu Z. Yu Y. Shen W. H. (2014). *Arabidopsis* AL PHD-PRC1 complexes promote seed germination through H3K4me3-to-H3K27me3 chromatin state switch in repression of seed developmental genes. *PLoS Genet.* 10:e1004091. 10.1371/journal.pgen.1004091 24465219 PMC3900384

[B90] Montalbán I. A. Moncaleán P. (2018). “*Pinus radiata* (D. Don) somatic embryogenesis,” in *Step Wise Protocols for Somatic Embryogenesis of Important Woody Plants*, Vol. I, eds Jain S. M. Gupta P. (Cham: Springer), 1–11. 10.1007/978-3-319-89483-6_1

[B91] Mozgová I. Muñoz-Viana R. Hennig L. (2017). PRC2 represses hormone-induced somatic embryogenesis in vegetative tissue of *Arabidopsis thaliana*. *PLoS Genet.* 13:e1006562. 10.1371/journal.pgen.1006562 28095419 PMC5283764

[B92] Negin B. Shemer O. Sorek Y. Williams L. E. (2017). Shoot stem cell specification in roots by the WUSCHEL transcription factor. *PLoS One* 12:e0176093. 10.1371/journal.pone.0176093 28445492 PMC5405954

[B93] Nic-Can G. I. Avilez-Montalvo J. R. Avilez-Montalvo R. N. Márquez-López R. E. Mellado-Mojica E. Galaz-Ávalos R. M. (2016). “The relationship between stress and somatic embryogenesis,” in *Somatic Embryogenesis. Fundamental Aspects and Applications*, eds Loyola-Vargas V. M. Ochoa-Alejo N. (Cham: Springer), 151–170. 10.1007/978-3-319-33705-0_9

[B94] Nic-Can G. I. López-Torres A. Barredo-Pool F. A. Wrobel K. Loyola-Vargas V. M. Rojas-Herrera R. (2013). New insights into somatic embryogenesis: *LEAFY COTYLEDON1*, *BABY BOOM1* and *WUSCHEL-RELATED HOMEOBOX4* are epigenetically regulated in *Coffea canephora*. *PLoS One* 8:e72160. 10.1371/journal.pone.0072160 23977240 PMC3748027

[B95] Nic-Can G. I. Loyola-Vargas V. M. (2016). “The role of the auxins during somatic embryogenesis,” in *Somatic Embryogenesis. Fundamental Aspects and Applications*, eds Loyola-Vargas V. M. Ochoa-Alejo N. (Cham: Springer), 171–181. 10.1007/978-3-319-33705-0_10

[B96] Noceda C. Salaj T. Pérez M. Viejo M. Cañal J. Salaj J. (2009). DNA methylation and decrease on free polyamines is associated with the embryogenic capacity of *Pinus nigra* Arn. Cell culture. *Trees* 23 1285–1293. 10.1007/s00468-009-0370-8

[B97] Nolan K. E. Irwanto R. R. Rose R. J. (2003). Auxin up-regulates *MtSERK1* expression in both *Medicago truncatula* root-forming and embryogenic cultures. *Plant Physiol.* 133 218–230. 10.1104/pp.103.020917 12970488 PMC196599

[B98] Novák O. Ljung K. (2017). Zooming in on plant hormone analysis: tissue- and cell-specific approaches. *Annu. Rev. Plant Biol.* 68 323–348. 10.1146/annurev-arplant-042916-040812 28226234

[B99] Nowak K. Gaj M. D. (2016). “Transcription factors in the regulation of somatic embryogenesis,” in *Somatic Embryogenesis: Fundamental Aspects and Applications*, eds Loyola-Vargas V. M. Ochoa-Alejo N. (Cham: Springer International Publishing), 53–79. 10.1007/978-3-319-33705-0_5

[B100] Ohme-Takagi M. Shinshi H. (1995). Ethylene-inducible DNA binding proteins that interect with an ethylene-responsive element. *Plant Cell* 7 173–182. 10.1105/tpc.7.2.173 7756828 PMC160773

[B101] Ooi S. E. Choo C. N. Ishak Z. Ong-Abdullah M. (2012). A candidate auxin-responsive expression marker gene, *EgIAA9*, for somatic embryogenesis in oil palm (*Elaeis guineensis* Jacq.). *Plant Cell Tissue Organ Cult.* 110 201–212. 10.1007/s11240-012-0143-8

[B102] Pandey D. K. Chaudhary B. (2014). Role of plant somatic embryogenesis receptor kinases (SERKs) in cell-to-embryo transitional activity: key at novel assorted structural subunits. *Am. J. Plant Sci.* 5 3177–3193. 10.4236/ajps.2014.521334

[B103] Passarinho P. Ketelaar T. Xing M. van Arkel J. Maliepaard C. Hendriks M. (2008). BABY BOOM target genes provide diverse entry points into cell proliferation and cell growth pathways. *Plant Mol. Biol.* 68 225–237. 10.1007/s11103-008-9364-y 18663586

[B104] Pelletier J. M. Kwong R. W. Park S. Le B. H. Baden R. Cagliari A. (2017). LEC1 sequentially regulates the transcription of genes involved in diverse developmental processes during seed development. *Proc. Natl. Acad. Sci. U.S.A.* 114 E6710–E6719. 10.1073/pnas.1707957114 28739919 PMC5559047

[B105] Pencik A. Tureková V. Paulisiç S. Rolcìk J. Strnad M. Mihaljevic S. (2015). Ammonium regulates embryogenic potential in *Cucurbita pepo* through pH-mediated changes in endogenous auxin and abscisic acid. *Plant Cell Tissue Organ Cult.* 122 89–100. 10.1007/s11240-015-0752-0

[B106] Pérez-Núñez M. T. Souza R. Sáenz L. Chan J. L. Zúñiga-Aguilar J. J. Oropeza C. (2009). Detection of a SERK-like gene in coconut and analysis of its expression during the formation of embryogenic callus and somatic embryos. *Plant Cell Rep.* 28 11–19. 10.1007/s00299-008-0616-8 18818928

[B107] Pérez-Pascual D. Jiménez-Guillen D. Villanueva-Alonzo H. Souza-Perera R. Godoy-Hernández G. Zúñiga-Aguilar J. J. (2018). Ectopic expression of the *Coffea canephora* SERK1 homologue induced differential transcription of genes involved in auxin metabolism and in the developmental control of embryogenesis. *Physiol. Plant.* 163 530–551. 10.1111/ppl.12709 29607503

[B108] Pila Quinga L. A. Heringer A. S. Pacheco de Freitas Fraga H. do Nascimento Vieira L. Silveira V. Steinmacher D. A. (2018). Insights into the conversion potential of *Theobroma cacao* L. somatic embryos using quantitative proteomic analysis. *Sci. Hortic.* 229 65–76. 10.1016/j.scienta.2017.10.005

[B109] Pila Quinga L. A. Pacheco de Freitas Fraga H. do Nascimento Vieira L. Guerra M. P. (2017). Epigenetics of long-term somatic embryogenesis in *Theobroma cacao* L.: DNA methylation and recovery of embryogenic potential. *Plant Cell Tissue Organ Cult.* 131 295–305. 10.1007/s11240-017-1284-6

[B110] Pilarska M. Malec P. Salaj J. Bartnicki F. Konieczny R. (2016). High expression of *SOMATIC EMBRYOGENESIS RECEPTOR-LIKE KINASE* coincides with initiation of various developmental pathways in *in vitro* culture of *Trifolium nigrescens*. *Protoplasma* 253 345–355. 10.1007/s00709-015-0814-5 25876517 PMC4783438

[B111] Quiroz-Figueroa F. R. Méndez-Zeel M. Sánchez-Teyer F. Rojas-Herrera R. Loyola-Vargas V. M. (2002). Differential gene expression in embryogenic and non-embryogenic clusters from cell suspension cultures of *Coffea arabica* L. *J. Plant Physiol.* 159 1267–1270. 10.1078/0176-1617-00878

[B112] Quiroz-Figueroa F. R. Monforte-González M. Galaz-Ávalos R. M. Loyola-Vargas V. M. (2006). “Direct somatic embryogenesis in *Coffea canephora*,” in *Plant Cell Culture Protocols*, eds Loyola-Vargas V. M. Vázquez-Flota F. A. (Totowa, NJ: Humana Press), 111–117. 10.1385/1-59259-959-1:111 16673910

[B113] Radoeva T. Weijers D. (2014). A roadmap to embryo identity in plants. *Trends Plant Sci.* 19 709–716. 10.1016/j.tplants.2014.06.009 25017700

[B114] Reinert J. Tazawa M. Semenoff S. (1967). Nitrogen compounds as factors of the embryogenesis *in vitro*. *Nature* 216 1215–1216. 10.1038/2161215a0 6076069

[B115] Rensing S. A. Lang D. Schumann E. Reski R. Hohe A. (2005). EST sequencing from embryogenic *Cyclamen persicum* cell cultures identifies a high proportion of transcripts homologous to plant genes involved in somatic embryogenesis. *J. Plant Growth Regul.* 24 102–115. 10.1007/s00344-005-0033-y

[B116] Riechmann J. L. Heard J. Martin G. Reuber L. Jiang C. Z. Keddie J. (2000). *Arabidopsis* transcription factors: genome-wide comparative analysis among eukaryotes. *Science* 290 2105–2110. 10.1126/science.290.5499.2105 11118137

[B117] Rosenquist M. Sehnke P. Ferl R. J. Sommarin M. Larsson C. (2000). Evolution of the 14-3-3 protein family: does the large number of isoforms in multicellular organisms reflect functional specificity? *J. Mol. Evol.* 51 446–458. 10.1007/s002390010107 11080367

[B118] Salo H. M. Sarjala T. Jokela A. Häggman H. Vuosku J. (2016). Moderate stress responses and specific changes in polyamine metabolism characterize Scots pine somatic embryogenesis. *Tree Physiol.* 36 392–402. 10.1093/treephys/tpv136 26786537 PMC4885945

[B119] Santa-Catarina C. de Oliveira R. R. Cutri L. Floh E. I. Dornelas M. C. (2012). WUSCHEL-related genes are expressed during somatic embryogenesis of the basal angiosperm *Ocotea catharinensis* Mez. (Lauraceae). *Trees* 26 493–501. 10.1007/s00468-011-0610-6

[B120] Santos D. Fevereiro P. (2002). Loss of DNA methylation affects somatic embryogenesis in *Medicago truncatula*. *Plant Cell Tissue Organ Cult.* 70 155–161. 10.1023/A:1016369921067

[B121] Schmidt E. D. L. Guzzo F. Toonen M. A. J. De Vries S. C. (1997). A leucine-rich repeat containing receptor-like kinase marks somatic plant cells competent to form embryos. *Development* 124 2049–2062. 9169851 10.1242/dev.124.10.2049

[B122] Schoonheim P. J. Sinnige M. P. Casaretto J. A. Veiga H. Bunney T. D. Quatrano R. S. (2007). 14-3-3 adaptor proteins are intermediates in ABA signal transduction during barley seed germination. *Plant J.* 49 289–301. 10.1111/j.1365-313X.2006.02955.x 17241451

[B123] Sharma S. K. Millam S. Hedley P. E. McNicol J. Bryan G. J. (2008). Molecular regulation of somatic embryogenesis in potato: an auxin led perspective. *Plant Mol. Biol.* 68 185–201. 10.1007/s11103-008-9360-2 18553172

[B124] Shimada T. Hirabayashi T. Endo T. Fujii H. Kita M. Omura M. (2005). Isolation and characterization of the somatic embryogenesis receptor-like kinase gene homologue (CitSERK1) from *Citrus unshiu* Marc. *Sci. Hortic.* 103 233–238. 10.1016/j.scienta.2004.07.005

[B125] Shiota H. Satoh R. Watabe K. Harada H. Kamada H. (1998). *C-AB13*, the carrot homologue of the *Arabidopsis AB13*, is expressed during both zygotic and somatic embryogenesis and functions in the regulation of embryo-specific ABA-inducible genes. *Plant Cell Physiol.* 39 1184–1193. 10.1093/oxfordjournals.pcp.a029319 9891417

[B126] Silva A. T. Barduche D. Do Livramento K. G. Paiva L. V. (2015). A putative BABY BOOM-like gene (CaBBM) is expressed in embryogenic calli and embryogenic cell suspension culture of *Coffea arabica* L. *In Vitro Cell. Dev. Biol. Plant* 51 93–101. 10.1007/s11627-014-9643-z

[B127] Singh A. Khurana P. (2017). Ectopic expression of *Triticum aestivum SERK* genes (*TaSERKs*) control plant growth and development in *Arabidopsis*. *Sci. Rep.* 7:12368. 10.1038/s41598-017-10038-1 28959050 PMC5620050

[B128] Singh P. Sinha A. K. (2017). “Interplay between auxin and cytokinin and its impact on mitogen activated protein kinase (MAPK),” in *Auxins and Cytokinins in Plant Biology: Methods and Protocols*, eds Dandekar T. Naseem M. (New York, NY: Springer), 93–100. 10.1007/978-1-4939-6831-2_7 28265990

[B129] Singla B. Khurana J. P. Khurana P. (2009). Structural characterization and expression analysis of the SERK/SERL gene family in rice (*Oryza sativa*). *Int. J. Plant Genomics* 2009:539402. 10.1155/2009/539402 19756234 PMC2742738

[B130] Smertenko A. Bozhkov P. V. (2014). Somatic embryogenesis: life and death processes during apical-basal patterning. *J. Exp. Bot.* 65 1343–1360. 10.1093/jxb/eru005 24622953

[B131] Somleva M. N. Schmidt E. D. L. De Vries S. C. (2000). Embryogenic cells in *Dactylis glomerata* L. (Poaceae) explants identified by cell tracking and by SERK expression. *Plant Cell Rep.* 19 718–726. 10.1007/s002999900169 30754811

[B132] Somssich M. Je B. I. Simon R. Jackson D. (2016). CLAVATA-WUSCHEL signaling in the shoot meristem. *Development* 143 3238–3248. 10.1242/dev.133645 27624829

[B133] Srinivasan C. Liu Z. Heidmann I. Jaya E. Fukoaka H. Joose R. (2007). Heterologous expression of the BABY BOOM AP2/ERF transcription factor enhances the regeneration capacity of tabacco (*Nicotiana tabacum* L.). *Planta* 225 341–351. 10.1007/s00425-006-0358-1 16924539

[B134] Stasolla C. Kong L. Yeung E. C. Thorpe T. A. (2002). Maturation of somatic embryos in conifers: morphogenesis, physiology, biochemistry, and molecular biology. *In Vitro Cell. Dev. Biol. Plant* 38 93–105. 10.1079/IVP2001262

[B135] Steiner N. Santa-Catarina C. Guerra M. Cutri L. Dornelas M. Floh E. (2012). A gymnosperm homolog of SOMATIC EMBRYOGENESIS RECEPTOR-LIKE KINASE-1 (*SERK1*) is expressed during somatic embryogenesis. *Plant Cell Tissue Organ Cult.* 109 41–50. 10.1007/s11240-011-0071-z

[B136] Stone S. L. Kwong L. W. Yee K. M. Pelletier J. Lepiniec L. Fischer R. L. (2001). Leafy cotyledon encodes a B3 domain transcription factor that induces embryo development. *Proc. Natl. Acad. Sci. U.S.A.* 98 11806–11811. 10.1073/pnas.201413498 11573014 PMC58812

[B137] Su Y. H. Zhao X. Y. Liu Y. B. Zhang C. L. O’Neill S. D. Zhang X. S. (2009). Auxin-induced *WUS* expression is essential for embryonic stem cell renewal during somatic embryogenesis in Arabidopsis. *Plant J.* 59 448–460. 10.1111/j.1365-313X.2009.03880.x 19453451 PMC2788036

[B138] Tanaka M. Kikuchi A. Kamada H. (2008). The Arabidopsis histone deacetylases HDA6 and HDA19 contribute to the repression of embryonic properties after germination. *Plant Physiol.* 146 149–161. 10.1104/pp.107.111674 18024558 PMC2230551

[B139] Tchorbadjieva M. (2016). “Advances in proteomics of somatic embryogenesis,” in *Somatic Embryogenesis in Ornamentals and Its Applications*, ed. Mujib A. (New Delhi: Springer), 67–90. 10.1007/978-81-322-2683-3_5

[B140] Thakare D. Tang W. Hill K. Perry S. E. (2008). The MADS-domain transcriptional regulator AGAMOUS-Like 15 promotes somatic embryo development in *Arabidopsis* and soybean. *Plant Physiol.* 146 1663–1672. 10.1104/pp.108.115832 18305206 PMC2287341

[B141] Thomas C. Meyer D. Himber C. Steinmetz A. (2004). Spatial expression of a sunflower SERK gene during induction of somatic embryogenesis and shoot organogenesis. *Plant Physiol. Biochem.* 42 35–42. 10.1016/j.plaphy.2003.10.008 15061082

[B142] Toonen M. A. J. Hendriks T. Schmidt E. D. L. Verhoeven H. A. Van Kammen A. De Vries S. C. (1994). Description of somatic-embryo-forming single cells in carrot suspension cultures employing video cell tracking. *Planta* 194 565–572. 10.1007/BF00714471

[B143] Trontin J. F. Klimaszewska K. Morel A. Hargreaves C. Lelu-Walter M. A. (2016). “Molecular aspects of conifer zygotic and somatic embryo development: a review of genome-wide approaches and recent insights,” in *In Vitro Embryogenesis in Higher Plants*, eds Germanà M. A. Lambardi M. (New York, NY: Springer), 167–207. 10.1007/978-1-4939-3061-6_8 26619863

[B144] Tvorogova V. E. Lutova L. A. (2018). Genetic regulation of zygotic embryogenesis in angiosperm plants. *Russ. J. Plant Physiol.* 65 1–14. 10.1134/S1021443718010107

[B145] Uddenberg D. Valladares S. Abrahamsson M. Sundström J. F. Sundás-Larsson A. Von Arnold S. (2011). Embryogenic potential and expression of embryogenesis-related genes in conifers are affected by treatment with a histone deacetylase inhibitor. *Planta* 234 527–539. 10.1007/s00425-011-1418-8 21541665 PMC3162143

[B146] Us-Camas R. Rivera-Solís G. Duarte-Aké F. De-la-Peña C. (2014). *In vitro* culture: an epigenetic challenge for plants. *Plant Cell Tissue Organ Cult.* 118 187–201. 10.1007/s11240-014-0482-8

[B147] Vale Ede M. Heringer A. S. Barroso T. Ferreira A. T. da Costa M. N. Perales J. E. A. (2014). Comparative proteomic analysis of somatic embryo maturation in *Carica papaya* L. *Proteome Sci.* 12:37. 10.1186/1477-5956-12-37 25076862 PMC4115220

[B148] Waki T. Hiki T. Watanabe R. Hashimoto T. Nakajima K. (2011). The *Arabidopsis* RWP-RK protein RKD4 triggers gene expression and pattern formation in early embryogenesis. *Curr. Biol.* 21 1277–1281. 10.1016/j.cub.2011.07.001 21802301

[B149] Walker K. A. Sato S. J. (1981). Morphogenesis in callus tissue of *Medicago sativa*: the role of ammonium ion in somatic embryogenesis. *Plant Cell Tissue Organ Cult.* 1 109–121. 10.1007/BF02318910

[B150] West M. A. L. Harada J. J. (1993). Embryogenesis in higher plants: an overview. *Plant Cell* 5 1361–1369. 10.1105/tpc.5.10.1361 12271035 PMC160368

[B151] Winkelmann T. (2016). “Somatic versus zygotic embryogenesis: learning from seeds,” in *In Vitro Embryogenesis in Higher Plants*, eds Germanà M. A. Lambardi M. (New York, NY: Springer), 25–46. 10.1007/978-1-4939-3061-6_2 26619857

[B152] Wójcikowska B. Gaj M. D. (2017). Expression profiling of *AUXIN RESPONSE FACTOR* genes during somatic embryogenesis induction in Arabidopsis. *Plant Cell Rep.* 36 843–858. 10.1007/s00299-017-2114-3 28255787 PMC5486788

[B153] Xiao Y. Chen Y. Ding Y. Wu J. Wang P. Yu Y. (2018). Effects of *GhWUS* from upland cotton (*Gossypium hirsutum* L.) on somatic embryogenesis and shoot regeneration. *Plant Sci.* 270 157–165. 10.1016/j.plantsci.2018.02.018 29576069

[B154] Xu M. Li X. Korban S. (2004). DNA-methylation alterations and exchanges during *in vitro* cellular differentiation in rose (*Rosa hybrida* L.). *Theor. Appl. Genet.* 109 899–910. 10.1007/s00122-004-1717-6 15221146

[B155] Yadav R. K. Perales M. Gruel J. Girke T. Jonsson H. Reddy G. V. (2011). WUSCHEL protein movement mediates stem cell homeostasis in the *Arabidopsis* shoot apex. *Genes Dev.* 25 2025–2030. 10.1101/gad.17258511 21979915 PMC3197201

[B156] Yakovlev I. A. Carneros E. Lee Y. Olsen J. E. Fossdal C. G. (2016). Transcriptional profiling of epigenetic regulators in somatic embryos during temperature induced formation of an epigenetic memory in Norway spruce. *Planta* 243 1237–1249. 10.1007/s00425-016-2484-8 26895338

[B157] Yamamoto N. Kobayashi H. Togashi T. Mori Y. Kikuchi K. Kuriyama K. (2005). Formation of embryogenic cell clumps from carrot epidermal cells is suppressed by 5-azacytidine, a DNA methylation inhibitor. *J. Plant Physiol.* 162 47–54. 10.1016/j.jplph.2004.05.013 15700420

[B158] Yang X. Zhang X. (2010). Regulation of somatic embryogenesis in higher plants. *Crit. Rev. Plant Sci.* 29 36–57. 10.1080/07352680903436291

[B159] Yang X. Zhang X. Yuan D. Jin F. Zhang Y. Xu J. (2012). Transcript profiling reveals complex auxin signalling pathway and transcription regulation involved in dedifferentiation and redifferentiation during somatic embryogenesis in cotton. *BMC Plant Biol.* 12:110. 10.1186/1471-2229-12-110 22817809 PMC3483692

[B160] Yao Y. Du Y. Jiang L. Liu J. Y. (2007). Molecular analysis and expression patterns of the 14-3-3 gene family from *Oryza sativa*. *J. Biochem. Mol. Biol.* 40 349–357. 10.5483/BMBRep.2007.40.3.349 17562286

[B161] Zhai L. Xu L. Wang Y. Zhu X. Feng H. Li C. (2016). Transcriptional identification and characterization of differentially expressed genes associated with embryogenesis in radish (*Raphanus sativus* L.). *Sci. Rep.* 6:21652. 10.1038/srep21652 26902837 PMC4763228

[B162] Zhang T. Q. Lian H. Zhou C. M. Xu L. Jiao Y. Wang J. W. (2017). A two-step model for *de novo* activation of WUSCHEL during plant shoot regeneration. *Plant Cell* 29 1073–1087. 10.1105/tpc.16.00863 28389585 PMC5466026

[B163] Zhang Y. Clemens A. Maximova S. N. Guiltinan M. J. (2014). The *Theobroma cacao* B3 domain transcription factor TcLEC2 plays a duel role in control of embryo development and maturation. *BMC Plant Biol.* 14:106. 10.1186/1471-2229-14-106 24758406 PMC4021495

[B164] Zhang Z. T. Zhou Y. Li Y. Shao S. Q. Li B. Y. Shi H. Y. (2010). Interactome analysis of the six cotton 14-3-3s that are preferentially expressed in fibres and involved in cell elongation. *J. Exp. Bot.* 61 3331–3344. 10.1093/jxb/erq155 20519337 PMC2905198

[B165] Zhao J. Wang B. Wang X. Zhang Y. Dong M. Zhang J. (2015). iTRAQ-based comparative proteomic analysis of embryogenic and non-embryogenic tissues of Prince Rupprecht’s larch (*Larix principis-rupprechtii* Mayr). *Plant Cell Tissue Organ Cult.* 120 655–669. 10.1007/s11240-014-0633-y

[B166] Zhao P. Begcy K. Dresselhaus T. Sun M. X. (2017). Does early embryogenesis in eudicots and monocots involve the same mechanism and molecular players? *Plant Physiol.* 173 130–142. 10.1104/pp.16.01406 27909044 PMC5210740

[B167] Zheng W. Zhang X. Yang Z. Wu J. Li F. Duan L. (2014). AtWuschel promotes formation of the embryogenic callus in *Gossypium hirsutum*. *PLoS One* 9:e87502. 10.1371/journal.pone.0087502 24498119 PMC3909107

[B168] Zhou T. Yang X. Guo K. Deng J. Xu J. Gao W. (2016). ROS homeostasis regulates somatic embryogenesis via the regulation of auxin signaling in cotton. *Mol. Cell. Proteomics* 15 2108–2124. 10.1074/mcp.M115.049338 27073181 PMC5083107

[B169] Zuo J. Niu Q. W. Frugis G. Chua N. H. (2002). The WUSCHEL gene promotes vegetative-to-embryonic transition in *Arabidopsis*. *Plant J.* 30 349–359. 10.1046/j.1365-313X.2002.01289.x 12000682

