# Supplementary material for: Signaling Overview of Plant Somatic Embryogenesis
Source: Front Plant Sci. 2019 Feb 7;10:77. doi: 10.3389/fpls.2019.00077 (PMC6375091; doi:10.3389/fpls.2019.00077)
Supplement: Supplementary file 1 [file Table_1.DOCX]

| **Transcription factor** | **Gymnosperm** | **Angiosperm** | |
| --- | --- | --- | --- |
|  |  | **Monocotyledons** | **Dicotyledons** |
| Aba insensitive 3 (ABI 3) | *Picea abies*  *Picea glauca*  *Pinus sylvestris* |  | *Arabidopsis thaliana*  *Daucus carota* |
| Agamous-like-15 (AGL15) | *Picea glauca* |  | *Arabidopsis thaliana*  *Brassica napus*  *Gossypium hirsutum* |
| Auxin response factor (ARF) |  | *Brachypodium distachyon* | *Arabidopsis thaliana*  *Carica papaya*  *Vitis vinifera* |
| Baby boom 1 (BBM1) | *Larix decidua*  *Picea glauca* |  | *Arabidopsis thaliana*  *Theobroma cacao* |
| Cup-shaped cotyledons (CUC) | *Araucaria angustifolia* |  | *Arabidopsis thaliana*  *Solanum lycopersicum* |
| Fusca 3 (FUS 3) |  |  | *Arabidopsis thaliana*  *Daucus carota*  *Medicago truncatula* |
| Leafy cotyledon (LEC) | *Larix decidua*  *Pinus contorta*  *Pinus sylvestris* | *Zea mays* | *Arabidopsis thaliana*  *Helianthus annus*  *Daucus carota* |
| Somatic embryogenesis receptor-like kinase 1 (SERK 1) | *Araucaria angustifolia*  *Picea glauca* | *Oryza sayiva* | *Arabidospsis thaliana*  *Coffea canephora*  *Daucus carota* |
| Viviparous 1 (VP 1) | *Picea abies*  *Picea glauca*  *Pinus sylvestris* |  | *Arabidospsis thaliana*  *Daucus carota*  *Quercus suber* |
| Wus relatet Homeobox2 (WOX 2) | *Araucaria angustifolia*  *Larix decidua*  *Picea abies* |  | *Arabidopsis thaliana*  *Coffea canephora*  *Vitis vinifera* |
| Wuschel (WUS) | *Picea abies*  *Picea glauca* |  | *Arabidopsis thaliana*  *Coffea canephora*  *Medicago truncatula* |

Table S1. Some TFs identified in plants belonging to different phylogenetic groups.
