# Supplementary material for: Signaling Overview of Plant Somatic Embryogenesis
Source: Front Plant Sci. 2019 Feb 7;10:77. doi: 10.3389/fpls.2019.00077 (PMC6375091; doi:10.3389/fpls.2019.00077)
Supplement: Supplementary file 2 [file Table_2.DOCX]

Table S2. Description of the identity of each gene showed in the interactome.

|  | **AUXIN** |
| --- | --- |
| **ARF10** | Auxin response factor 10; Auxin response factors (ARFs) are transcriptional factors that binds specifically to the DNA sequence 5’-TGTCTC-3’ found in the auxin-responsive promoter elements (AuxREs). Could act as transcriptional activator or repressor. Formation of heterodimers with Aux/IAA proteins may alter their ability to modulate early auxin response genes expression (693 aa) |
| **ARF16** | Auxin response factor 16; Auxin response factors (ARFs) are transcriptional factors that binds specifically to the DNA sequence 5’-TGTCTC-3’ found in the auxin-responsive promoter elements (AuxREs). Could act as transcriptional activator or repressor. Formation of heterodimers with Aux/IAA proteins may alter their ability to modulate early auxin response genes expression (670 aa) |
| **ARF19** | Auxin response factor 19; Auxin response factors (ARFs) are transcriptional factors that binds specifically to the DNA sequence 5’-TGTCTC-3’ found in the auxin-responsive promoter elements (AuxREs). Could act as transcriptional activator or repressor. Formation of heterodimers with Aux/IAA proteins may alter their ability to modulate early auxin response genes expression. Involved in ethylene responses. Regulates lateral root formation through direct regulation of LBD16 and/or LBD29. Functionally redundant with ARF7 (1086 aa) |
| **ARF2** | Auxin response factor 2; Auxin response factors (ARFs) are transcriptional factors that binds specifically to the DNA sequence 5’-TGTCTC-3’ found in the auxin-responsive promoter elements (AuxREs). Could act as transcriptional activator or repressor. Formation of heterodimers with Aux/IAA proteins may alter their ability to modulate early auxin response genes expression. Promotes flowering, stamen development, floral organ abscission and fruit dehiscence. Functions independently of ethylene and cytokinin response pathways. May act as a repressor of cell division and organ growth (859 aa) |
| **ARF4** | Auxin response factor 4; Auxin response factors (ARFs) are transcriptional factors that binds specifically to the DNA sequence 5’-TGTCTC-3’ found in the auxin-responsive promoter elements (AuxREs). Could act as transcriptional activator or repressor. Formation of heterodimers with Aux/IAA proteins may alter their ability to modulate early auxin response genes expression (788 aa) |
| **ARF6** | Auxin response factor 6; Auxin response factors (ARFs) are transcriptional factors that binds specifically to the DNA sequence 5’-TGTCTC-3’ found in the auxin-responsive promoter elements (AuxREs). Seems to act as transcriptional activator. Formation of heterodimers with Aux/IAA proteins may alter their ability to modulate early auxin response genes expression. Regulates both stamen and gynoecium maturation. Promotes jasmonic acid production. Partially redundant with ARF8 (935 aa) |
| **ARF8** | Auxin response factor 8; Auxin response factors (ARFs) are transcriptional factors that binds specifically to the DNA sequence 5’-TGTCTC-3’ found in the auxin-responsive promoter elements (AuxREs). Seems to act as transcriptional activator. Formation of heterodimers with Aux/IAA proteins may alter their ability to modulate early auxin response genes expression. Regulates both stamen and gynoecium maturation. Promotes jasmonic acid production. Partially redundant with ARF6. Involved in fruit initiation. Acts as an inhibitor to stop further carpel development in the absence of fertilizat [...] (811 aa) |
| **ARF9** | Auxin response factor 9; Auxin response factors (ARFs) are transcriptional factors that binds specifically to the DNA sequence 5’-TGTCTC-3’ found in the auxin-responsive promoter elements (AuxREs). Could act as transcriptional activator or repressor. Formation of heterodimers with Aux/IAA proteins may alter their ability to modulate early auxin response genes expression (638 aa) |
| **AXR4** | AUXIN RESISTANT 4; Required for the auxin influx facilitator AUX1 polar trafficking and its asymmetric localization within the plasma membrane. Not involved in the PIN proteins localization (473 aa) |
| **PIN1** | PIN-FORMED 1; Acts as a component of the auxin efflux carrier. Seems to be involved in the basipetal auxin transport. Mediates the formation of auxin gradient which is required to ensure correct organogenesis. Coordinated polar localization of PIN1 is directly regulated by the vesicle trafficking process and apical-basal PIN1 polarity also depends on the phosphorylation of conserved serine residues by PID kinase. The ARF-GEF protein GNOM is required for the correct recycling of PIN1 between the plasma membrane and endosomal compartments (622 aa) |
| **PIN3** | PIN-FORMED 3; Acts as a component of the auxin efflux carrier. Seems to be involved in the lateral auxin transport system and mediates tropic growth. Coordinated polar localization of PIN3 is directly regulated by the vesicle trafficking process (640 aa) |
| **PIN5** | PIN-FORMED 5; May act as a transmembrane component of the auxin efflux carrier (351 aa) |
| **PIN6** | PIN-FORMED 6; May act as a component of the auxin efflux carrier (570 aa) |
| **PIN8** | PIN-FORMED 8; May act as a component of the auxin efflux carrier (367 aa) |
| **TAA1** | Tryptophan aminotransferase; L-tryptophan aminotransferase involved in auxin (IAA) biosynthesis. Can convert L-tryptophan and pyruvate to indole-3- pyruvic acid (IPA) and alanine. Catalyzes the first step in IPA branch of the auxin biosynthetic pathway. Required for auxin production to initiate multiple change in growth in response to environmental and developmental cues. It is also active with phenylalanine, tyrosine, leucine, alanine, methionine and glutamine. Both TAA1 and TAR2 are required for maintaining proper auxin levels in roots, while TAA1, TAR1 and TAR2 are required for prop [...] (391 aa) |
| **TAR2** | Tryptophan aminotransferase related 2; Involved in auxin production. Both TAA1 and TAR2 are required for maintaining proper auxin levels in roots, while TAA1, TAR1 and TAR2 are required for proper embryo patterning. Involved in the maintenance of the root stem cell niches (440 aa) |
| **YUC10** | YUCCA 10; Involved in auxin biosynthesis (383 aa) |
| **YUC11** | Flavin-binding monooxygenase family protein; Involved in auxin biosynthesis (391 aa) |
| **YUC2** | YUCCA2; Involved in auxin biosynthesis. Converts the indole-3- pyruvic acid (IPA) produced by the TAA family to indole-3-acetic acid (IAA). Unable to use tryptamine (TAM) as substrate. Required for the formation of floral organs and vascular tissues. Belongs to the set of redundant YUCCA genes probably responsible for auxin biosynthesis in shoots (415 aa) |
| **YUC3** | YUCCA 3; Involved in auxin biosynthesis. Belongs to the set of redundant YUCCA genes probably responsible for auxin biosynthesis in roots (437 aa) |
| **YUC4** | YUCCA4; Involved in auxin biosynthesis. Both isoforms are catalitically active. Involved during embryogenesis and seedling development. Required for the formation of floral organs and vascular tissues. Belongs to the set of redundant YUCCA genes probably responsible for auxin biosynthesis in shoots (411 aa) |
| **YUC6** | YUCCA6; Involved in auxin biosynthesis via the indole-3-pyruvic acid (IPA) pathway. Also able to convert *in vitro* phenyl pyruvate (PPA) to phenyl acetic acid (PAA). Required for the formation of floral organs and vascular tissues. Belongs to the set of redundant YUCCA genes probably responsible for auxin biosynthesis in shoots (426 aa) |
| **YUC8** | YUCCA 8; Involved in auxin biosynthesis. Belongs to the set of redundant YUCCA genes probably responsible for auxin biosynthesis in roots (426 aa) |
|  | **CYTOKININ** |
| **ABCG14** | ATP-binding cassette 14 (648 aa) |
| **CKX1** | Cytokinin dehydrogenase 1; Catalyzes the oxidation of cytokinins, a family of N(6)- substituted adenine derivatives that are plant hormones, where the substituent is an isopentenyl group (575 aa) |
| **CKX3** | Cytokinin oxidase 3; Catalyzes the oxidation of cytokinins, a family of N(6)- substituted adenine derivatives that are plant hormones, where the substituent is an isopentenyl group (523 aa) |
| **CKX5** | Cytokinin oxidase 5; Catalyzes the oxidation of cytokinins, a family of N(6)- substituted adenine derivatives that are plant hormones, where the substituent is an isopentenyl group (540 aa) |
| **CKX7** | Cytokinin oxidase 7; Catalyzes the oxidation of cytokinins, a family of N(6)- substituted adenine derivatives that are plant hormones, where the substituent is an isopentenyl group (524 aa) |
| **ENT1** | Equilibrative nucleotide transporter 1; Nucleoside transporter involved in adenosine transport and required for nucleotide metabolism which influences growth and pollen germination. Has high affinity for adenosine when expressed in a heterologous system (yeast) (450 aa) |
| **IPT1** | Isopentenyltransferase 1; Involved in cytokinin biosynthesis. Catalyzes the transfer of an isopentenyl group from dimethylallyl diphosphate (DMAPP) to ATP, ADP and AMP. Adenine, adenosine, isopentenylpyrophosphate and 1-hydroxy-2-methyl-2-(E)-butenyl 4- diphosphate (HMBDP) are not used as substrates (357 aa) |
| **IPT2** | tRNAisopentenyltransferase 2; Catalyzes the transfer of a dimethylallyl group onto the adenine at position 37 in tRNAs that read codons beginning with uridine, leading to the formation of N6-(dimethylallyl)adenosine (i(6)A). Involved in the cis-type cytokinin biosynthesis (466 aa) |
| **IPT5** | Isopentenyltransferase 5; Involved in cytokinin biosynthesis. Catalyzes the transfer of an isopentenyl group from dimethylallyl diphosphate (DMAPP) to ATP and ADP (330 aa) |
| **IPT9** | Isopentenyltransferase 9; Catalyzes the transfer of a dimethylallyl group onto the adenine at position 37 in tRNAs that read codons beginning with uridine, leading to the formation of N6-(dimethylallyl)adenosine (i(6)A). Involved in the cis-type cytokinin biosynthesis (469 aa) |
| **LOG1** | LONELY GUY 1; Cytokinin-activating enzyme working in the direct activation pathway. Phosphoribohydrolase that converts inactive cytokinin nucleotides to the biologically active free-base forms (213 aa) |
| **LOG5** | LONELY GUY 5; Cytokinin-activating enzyme working in the direct activation pathway. Phosphoribohydrolase that converts inactive cytokinin nucleotides to the biologically active free-base forms (228 aa) |
| **LOG7** | LONELY GUY 7; Cytokinin-activating enzyme working in the direct activation pathway. Phosphoribohydrolase that converts inactive cytokinin nucleotides to the biologically active free-base forms (217 aa) |
| **LOG8** | LONELY GUY 8; Cytokinin-activating enzyme working in the direct activation pathway. Phosphoribohydrolase that converts inactive cytokinin nucleotides to the biologically active free-base forms (216 aa) |
| **PUP1** | Purine permease 1; Proton-coupled purine transporter mediating adenine and trans-zeatin uptake. Also able to transport caffeine and adenosine. May be involved in the uptake of cytokinin, caffeine and nicotine from the xylem sap into shoot tissues (356 aa) |
| **PUP10** | Purine permease 10 (390 aa) |
| **PUP11** | Purine permease 11 (379 aa) |
| **PUP3** | Purine permease 3; May be involved in transport of purine derivatives during pollen germination and tube elongation (351 aa) |
| **PUP4** | Purine permease 4 (382 aa) |
| **PUP5** | Purine permease 5 (361 aa) |
|  | **TRANSCRIPTION FACTOR** |
| **AG** | AGAMOUS; Probable transcription factor involved in the control of organ identity during the early development of flowers. Is required for normal development of stamens and carpels in the wild-type flower. Plays a role in maintaining the determinacy of the floral meristem. Acts as C class cadastral protein by repressing the A class floral homeotic genes like APETALA1. Forms a heterodimer via the K-box domain with either SEPALATTA1/AGL2, SEPALATTA2/AGL4, SEPALLATA3/AGL9 or AGL6 that could be involved in genes regulation during floral meristem development. Controls AHL21/GIK, a multifunct [...] (252 aa) |
| **AGL104** | AGAMOUS-like 104; Probable transcription factor that forms heterodimers with the MADS-box proteins AGL30 and AGL65 and is involved in the regulation of pollen maturation at the late stages of pollen development and pollen tube growth (335 aa) |
| **AGL12** | AGAMOUS-like 12; Probable transcription activator that regulates root development by controlling cell proliferation in root meristem. May mediate responses to auxin in the root. May act as promoter of the flowering transition through up-regulation of SOC, FT and LFY (211 aa) |
| **AGL15** | AGAMOUS-like 15; Transcription factor involved in the negative regulation of flowering, probably through the photoperiodic pathway. Acts as both an activator and a repressor of transcription. Binds DNA in a sequence-specific manner in large CArG motif 5’-CC (A/T)8 GG-3’. Participates probably in the regulation of programs active during the early stages of embryo development. Prevents premature perianth senescence and abscission, fruits development and seed desiccation. Stimulates the expression of at least DTA4, LEC2, FUS3, ABI3, AT4G38680/CSP2 and GRP2B/CSP4. Can enhance somatic embry [...] (268 aa) |
| **AGL16** | AGAMOUS-like 16; Probable transcription factor (240 aa) |
| **AGL19** | AGAMOUS-like 19; Probable transcription factor that promotes flowering, especially in response to vernalization by short periods of cold, in an FLC-inpedendent manner (219 aa) |
| **AGL31** | AGAMOUS-like 31; Probable transcription factor that prevents vernalization by short periods of cold. Acts as a floral repressor (196 aa) |
| **AGL61** | AGAMOUS-like 61; Probable transcription factor. Controls central cell differentiation during female gametophyte development (264 aa) |
| **AGL8** | AGAMOUS-like 8; Probable transcription factor that promotes early floral meristem identity in synergy with APETALA1 and CAULIFLOWER. Is required subsequently for the transition of an inflorescence meristem into a floral meristem. Seems to be partially redundant to the function of APETALA1 and CAULIFLOWER in the up-regulation of LEAFY. Is also required for normal pattern of cell division, expansion and differentiation during morphogenesis of the silique. Probably not required for fruit elongation but instead is required to prevent ectopic activity of IND (242 aa) |
| **AP1** | APETALA1; Transcription factor that promotes early floral meristem identity in synergy with LEAFY. Is required subsequently for the transition of an inflorescence meristem into a floral meristem. Is indispensable for normal development of sepals and petals in flowers. Regulates positively the B class homeotic proteins APETALA3 and PISTILLATA with the cooperation of LEAFY and UFO. Interacts with SEPALLATA3 or AP3/PI heterodimer to form complexes that could be involved in genes regulation during floral meristem development. Regulates positively AGAMOUS in cooperation with LEAFY. Displays [...] (256 aa) |
| **CLE13** | CLAVATA3/ESR-RELATED 13; Extracellular signal peptide that regulates cell fate. Represses root apical meristem maintenance (107 aa) |
| **CLV1** | CLAVATA 1; Involved in the detection of CLV3 and CLV3-like peptides, that act as extracellular signals regulating meristem maintenance. Acts with CLV3 as a ligand-receptor pair in a signal transduction pathway coordinating growth between adjacent meristematic regions and controlling the balance between meristem cell proliferation and differentiation (980 aa) |
| **CLV2** | Clavata 2; Involved in the perception of CLV3 and CLV3-like peptides, that act as extracellular signals regulating meristems maintenance. Involved in controlling the stem cell population size in shoot and root apical meristems, and during organ development. Promotes the formation of CLV1 multimers (720 aa) |
| **MP** | MONOPTEROS; Auxin response factors (ARFs) are transcriptional factors that binds specifically to the DNA sequence 5’-TGTCTC-3’ found in the auxin-responsive promoter elements (AuxREs). Seems to act as transcriptional activator. Formation of heterodimers with Aux/IAA proteins may alter their ability to modulate early auxin response genes expression. Mediates embryo axis formation and vascular tissues differentiation. Functionally redundant with ARF7. May be necessary to counteract AMP1 activity (902 aa) |
| **PRS** | PRESSED FLOWER; Probable transcription factor required to initiate organ founder cells in a lateral domain of shoot meristems. Involved in the lateral sepal axis-dependent development of flowers, probably by regulating the proliferation of L1 cells at the lateral region of flower primordia. Required for the formation of the margin cells of the first and second whorl organs (244 aa) |
| **SEP3** | SEPALLATA3; Probable transcription factor active in inflorescence development and floral organogenesis. Functions with SEPALLATA1/AGL2 and SEPALLATA2/AGL4 to ensure proper development of petals, stamens and carpels and to prevent the indeterminate growth of the flower meristem. Interacts with APETALA1, AGAMOUS or APETALA3/PISTILLATA to form complexes, that could be involved in genes regulation during floral meristem development (251 aa) |
| **SERK1** | Somatic embryogenesis receptor kinase 1; Dual specificity kinase acting on both serine/threonine- and tyrosine-containing substrates. Phosphorylates BRI1 on ’Ser- 887’ and CDC48 on at least one threonine residue and on ’Ser-41’. Confers embryogenic competence. Acts redundantly with SERK2 as a control point for sporophytic development controlling male gametophyte production. Involved in the brassinolide signaling pathway (625 aa) |
| **STK** | SEEDSTICK; Probable transcription factor (256 aa) |
| **WOX1** | WUSCHEL related homeobox 1; Transcription factor which may be involved in developmental processes (350 aa) |
| **WOX11** | WUSCHEL related homeobox 11 (297 aa) |
| **WOX11** | WUSCHEL related homeobox 11 (297 aa) |
| **WOX13** | WUSCHEL related homeobox 13; Transcription factor which may be involved in developmental processes (268 aa) |
| **WOX2** | WUSCHEL related homeobox 2; Probable transcription factor involved in embryonic patterning. Required for apical embryo development after fertilization. Its specific localization to the apical daughter cell of the zygote, while WOX8 is confined to the basal cell, suggests that the asymmetric division of the plant zygote separates determinants of apical and basal cell fates (260 aa) |
| **WOX4** | WUSCHEL related homeobox 4; Transcription factor which may be involved in developmental processes (251 aa) |
| **WOX5** | WUSCHEL related homeobox 5; Transcription factor, which may be involved in the specification and maintenance of the stem cells (QC cells) in the root apical meristem (RAM) (182 aa) |
| **WOX8** | WUSCHEL related homeobox 8; Probable transcription factor, which may be involved in embryonic patterning (PubMed-14711878). May be required for basal embryo development after fertilization (PubMed-14711878). Acts partially redundantly with STIP in promoting embryonic cell division and proliferation (PubMed-17706632). Promotes cotyledon boundary formation by maintaining the symmetry in CUC genes expression domains (PubMed-22827849) (325 aa) |
| **WUS** | WUSCHEL; Transcription factor that plays a central role during early embryogenesis, oogenesis and flowering, probably by regulating expression of specific genes. Required to specify stem cell identity in meristems, such as shoot apical meristem (SAM). May induce shoot stem cells activity in order to maintain the stem cell identity. Involved in the developmental root meristem. In shoot apices, it is sufficient to induce the expression of CLV3, a putative ligand of the CLV signaling pathway. Also required to sustain organogenesis in the floral meristem by contributing to the expression o [...] (292 aa) |
|  | **OTHER GENES INVOLVED** |
| **ABCG31** | ATP-binding cassette G31; May be a general defense protein (1426 aa) |
| **ABCG40** | ATP-binding cassette G40; May be a general defense protein (By similarity). Functions as a pump to exclude Pb(2+) ions and/or Pb(2+)- containing toxic compounds from the cytoplasm. Contributes to Pb(2+) ions resistance. Confers some resistance to the terpene sclareol (1423 aa) |
| **ABCG9** | ATP-binding cassette G9 (638 aa) |
| **AGL62** | AGAMOUS-like 62; Probable transcription factor. Required for suppression of cellularization and promotion of nuclear proliferation during early endosperm development. The FERTILIZATION-INDEPENDENT SEED (FIS) polycomb complex is required for suppression of ALG62 expression at the end of the syncytial phase of endosperm development (299 aa) |
| **AT1G02630** | Nucleoside transporter-like protein; May be involved in nucleoside transport (389 aa) |
| **AT1G34060** | Pyridoxal phosphate-dependent transferase domain-containing protein; Probable aminotransferase (463 aa) |
| **At1g59750** | Auxin response factor 1; Auxin response factors (ARFs) are transcriptional factors that binds specifically to the DNA sequence 5’-TGTCTC-3’ found in the auxin-responsive promoter elements (AuxREs). Seems to act as transcriptional repressor. Formation of heterodimers with Aux/IAA proteins may alter their ability to modulate early auxin response genes expression. Promotes flowering, stamen development, floral organ abscission and fruit dehiscence. Acts as repressor of IAA2, IAA3 and IAA7 (665 aa) |
| **At2g42590** | 14-3-3-like protein GF14 mu; Is associated with a DNA binding complex that binds to the G box, a well-characterized cis-acting DNA regulatory element found in plant genes (276 aa) |
| **At3g02520** | 14-3-3-like protein GF14 nu; Is associated with a DNA binding complex that binds to the G box, a well-characterized cis-acting DNA regulatory element found in plant genes (265 aa) |
| **AT5G21090** | Leucine-rich repeat-containing protein (218 aa) |
| **At5g65430** | General regulatory factor 8; Is associated with a DNA binding complex that binds to the G box, a well-characterized cis-acting DNA regulatory element found in plant genes. Involved in the regulation of nutrient metabolism (PubMed-22104211) (260 aa) |
| **CLE16** | CLAVATA3/ESR-RELATED 16; Extracellular signal peptide that regulates cell fate. Represses root apical meristem maintenance (103 aa) |
| **EIR1** | ETHYLENE INSENSITIVE ROOT 1; Acts as a component of the auxin efflux carrier. Seems to be involved in the root-specific auxin transport, and mediates the root gravitropism. Its particular localization suggest a role in the translocation of auxin towards the elongation zone (647 aa) |
| **ETT** | ETTIN; Auxin response factors (ARFs) are transcriptional factors that binds specifically to the DNA sequence 5’-TGTCTC-3’ found in the auxin-responsive promoter elements (AuxREs). Could act as transcriptional activator or repressor. Formation of heterodimers with Aux/IAA proteins may alter their ability to modulate early auxin response genes expression. Involved in the establishment or elaboration of tissue patterning during gynoecial development (608 aa) |
| **GF14** | General regulatory factor 2; Is associated with a DNA binding complex that binds to the G box, a well-characterized cis-acting DNA regulatory element found in plant genes (259 aa) |
| **GRF11** | General regulatory factor 11; Is associated with a DNA binding complex that binds to the G box, a well-characterized cis-acting DNA regulatory element found in plant genes (255 aa) |
| **GRF12** | General regulatory factor 12; Is associated with a DNA binding complex that binds to the G box, a well-characterized cis-acting DNA regulatory element found in plant genes (268 aa) |
